# Cardiac Structure and Function in Junior Athletes: A Systematic Review of Echocardiographic Studies

**DOI:** 10.31083/j.rcm2304129

**Published:** 2022-04-07

**Authors:** Heidi Weberruß, Tobias Engl, Lisa Baumgartner, Frauke Mühlbauer, Nerejda Shehu, Renate Oberhoffer-Fritz

**Affiliations:** ^1^Institute of Preventive Pediatrics, TUM Department of Sport and Health Sciences, Technical University of Munich, 80992 Munich, Germany; ^2^Department of Pediatric Cardiology and Congenital Heart Disease, German Heart Center Munich, Technical University of Munich, 80636 Munich, Germany

**Keywords:** athlete's heart, junior athletes, 2D transthoracic echocardiography, 2D speckle tracking echocardiography, cardiac remodelling

## Abstract

**Background:**

In young athletes, the level of competitiveness in sports is increasing, as well as frequency and intensity of exercise training. Adaptations of the cardiac system to this increased workload imposed by exercise has not yet been studied sufficiently. In adults, studies point towards a shift from the functional athlete’s heart towards pathological cardiac remodelling, with ventricular arrythmia and impaired cardiac function, that is exercise-related. This systematic review investigates cardiac adaptations to exercise in junior athletes compared to inactive controls.

**Methods:**

Three electronic databases (PubMed/Medline, ScienceDirect and Web of Science) were searched for studies assessing 2-dimensional transthoracic echocardiography (2D TTE) and 2-dimensional speckle tracking echocardiography (2D STE) parameters in junior athletes, aged 7–19 years, compared to inactive controls. Data was screened and extracted by two reviewers; study quality and risk of bias was assessed by three reviewers.

**Results:**

Eight out of 1460 studies met all inclusion criteria, with all studies reporting results on 2D TTE and six studies reporting results on 2D STE parameters in 540 (51 girls) junior athletes and 270 (18 girls) controls. There is evidence for structural cardiac adaptations of the left ventricle and both atria in junior athletes. Results regarding left ventricular function are controversial with a tendency to improved function in dynamic exercising athletes. Left ventricular mass and relative wall thickness point towards higher values in static exercising athletes.

**Conclusions:**

Cardiac adaptations to exercise occur in children and adolescents. These adaptations are more pronounced in structural left ventricular parameters. Functional parameters are preserved or slightly improved in junior athletes but not impaired by exercise.

## 1. Introduction

Young athletes performing sports on a competitive level practice between 10–20 hours a week at moderate to high intensities [1]. To keep up with the increased demands imposed on the body by intensive physical exercise, the cardiovascular system has to increase its capacity by a factor of 5–6 compared to moderate exercise [2]. Additionally, the level of competitiveness is increasing as well as training frequency, intensity, and demands that are placed into children’s training sessions [3, 4]. Several studies have reported cardiac remodelling in children and adolescents [5, 6, 7, 8]. Apparently, cardiac adaptation does not require the time span of a long professional training career.

To provide an overview of the current state of research regarding structural and functional cardiac adaptation in junior athletes, and to present results of these studies regarding the influence of exercise on the cardiovascular system, we performed this systematic review, searching electronic databases for studies investigating the cardiac structure and function in male and female junior athletes (7–19 years) compared to a non-active control group by two-dimensional transthoracic echocardiography (2D TTE) or 2D speckle tracking echocardiography (STE).

## 2. Materials and Methods

This review is in line with the PRISMA Statement [9].

**Search strategy and selection criteria**: We searched databases PubMed/Medline, ScienceDirect, and Web of Science. Inclusion criteria were (1) exercising or active children and/or adolescents, aged 7–19 years; (2) comparison of athletes with an inactive control group (CG); (3) performing 2-dimensional transthoracic echocardiography and/or 2-dimensional speckle tracking echocardiography. The exact search term was: ((children OR adolescents) AND (activ* OR trained OR exercise) AND (echocardiography or speckle tracking) AND (control group)). Only articles published in English were included. Review articles and meta-analyses were not considered, as well as articles including animal studies and the use of patients. Further exclusion criteria were: (1) not meeting our age criteria; (2) not exercising regularly/at a competitive level; (3) no inactive control-group; (4) other cardiac imaging methods. Results were screened by two researchers separately (HW and TE).

**Risk of bias assessment**: The risk of bias assessment for methods was performed according to an 11-item checklist for case-control studies [10]. To assess the risk of bias in study results a 12-item checklist was applied based on a recent review [4]. Three researchers (HW, LB, TE) screened the methods section and checked each study’s results. If no agreement could be found a consensual decision was made.

**Data extraction**: A standardized data extraction form was set up (TE) and cross-checked (HW). Where study data was unclear, authors of the corresponding publication were contacted.

**Quality assessment**: Study’s quality was assessed with the study quality assessment tool by the NIH National Blood, Heart, and Lung Institute [11]. Criteria were rated by three researchers (HW, LB, TE). If no agreement could be found a consensual decision was made.

## 3. Results

**Fig. 1.**
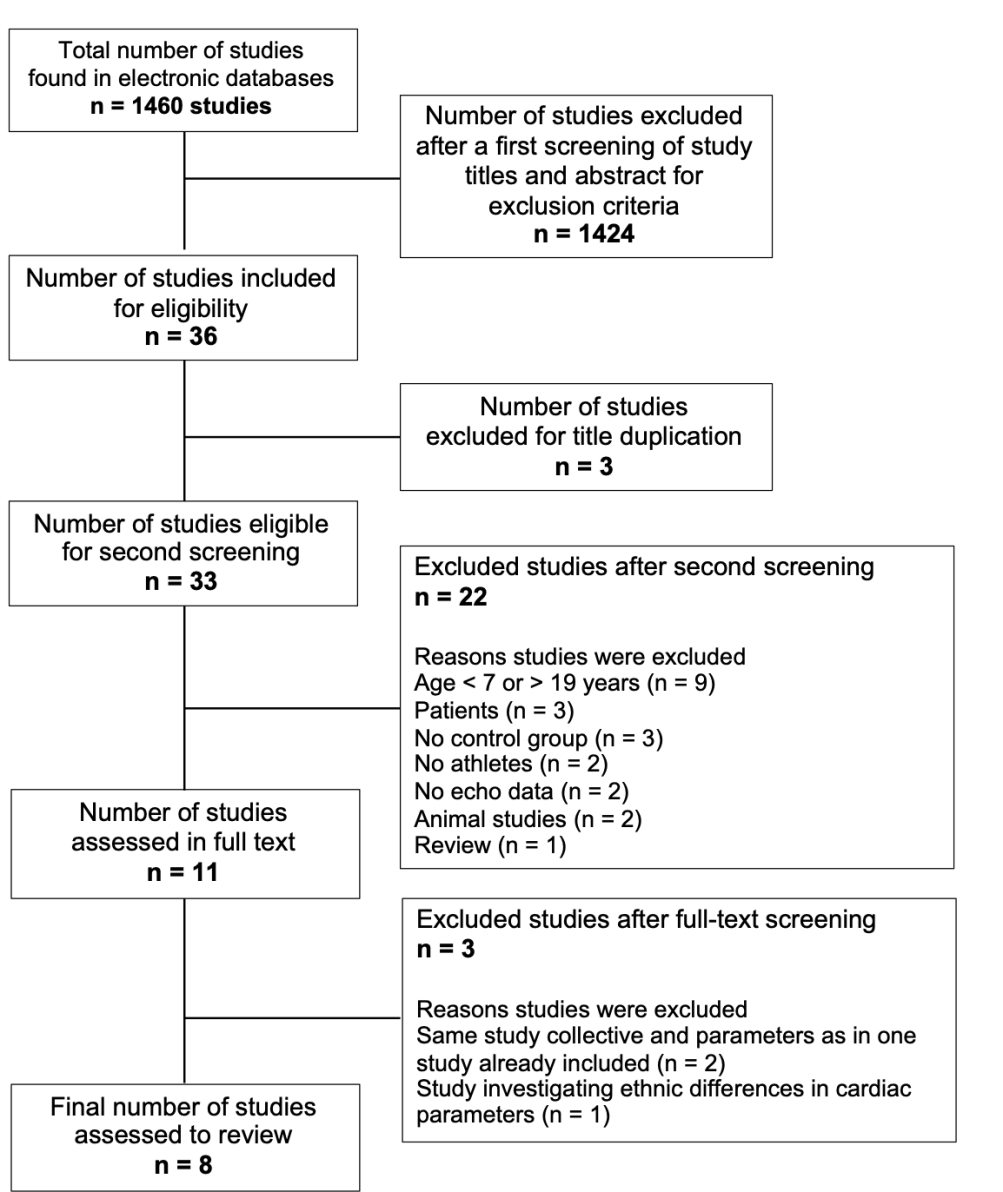
**Flow chart of the study selection process**.

In total, eight of 1460 studies met all inclusion criteria. Most of the studies (1424/97.7%) were excluded after the first screening. The majority of these studies did not deal with the matter of this review (43%), included patients (29%), did not meet our age criteria (16.5%), included animals (7%), or were recommendations or reviews (4%). Results of our search and reasons for excluding studies are shown in Fig. 1.

### 3.1 Risk of Bias Assessment

Regarding the risk of bias assessment for methods full agreement was met in 44% of cases. Regarding the risk of bias assessment in reporting results, the researchers fully agreed in 93% of cases (see **Supplementary Table 1** and **Supplementary Table 2**).

### 3.2 Quality Assessment

Five studies were rated to be of good quality [3, 12, 13, 14, 15], one as fair [16] and two studies as being of poor quality [17, 18]. Full agreement between the researchers was met in 60.6% and a majority agreement in 38.3% of the categories. Authors did not agree on 1 point (1.1%) (see **Supplementary Table 3**).

### 3.3 Study Groups Characteristics

Sample sizes varied from n = 44 to n = 300 participants [3, 12, 13, 14, 17]. Only two studies included female athletes [14, 16]. All studies included athletes performing predominantly dynamic (soccer and tennis) or mixed types of sports (basketball, running, cross-country skiing). Three studies included static types of sports [16, 17, 18]. A minimum training history of two years was required in four studies [14, 16, 17, 18]. Athletes in other studies trained for an average 4–6 years [12, 15, 18]. Training time per week varied from 2.5–3 hours [14, 16] up to 15 hours [17]. The control group’s activity level was <2 hours in most studies. An overview of the studies is given in **Supplementary Table 4**.

### 3.4 Anthropometry, Heart Rate and Blood Pressure

Anthropometric characteristics of study participants (age, body height, body mass, body surface area [BSA], body mass index [BMI]) as well as heart rate, systolic blood pressure [SBP], and diastolic blood pressure [DBP] are displayed in **Supplementary Table 5**.

### 3.5 Heart Rate and Blood Pressure

Four authors reported a significantly lower heart rate in athletes compared to controls (*p <* 0.05) [3, 13, 14, 17, 18]. Blood pressure results are inconsistent, with significantly lower results in athletes [3, 13], in dynamic-exercising athletes only [18] or only for DBP [15] or no significant differences in athletes vs. controls [12, 14, 17].

### 3.6 2D Transthoracic Echocardiography 

There was a huge variety of echocardiographic parameters and their methodological approach which made it difficult to compare these studies. Most authors focused on parameters regarding the left heart [12, 14, 15, 16, 17, 18] and two studies on the right heart’s structure and function [3, 13]. Results were categorized according to the heart’s structure and function.

#### 3.6.1 Left Ventricular Structure

Six studies focused on LV structure [12, 14, 15, 16, 17, 18]. As structural parameters are largely influenced by BSA [19, 20] only indexed parameters were compared: LV end-diastolic diameter (LVEDD), LV end-systolic diameter (LVESD), interventricular septal thickness (IVS), LV wall thickness (LVWT), LV posterior wall thickness (LVPWT), mean wall thickness (MWT), relative wall thickness (RWT), LVM, LV length, LV end-diastolic volume (LVEDV), and LV end-systolic volume (LVESV). LVEDD was significantly higher in athletes in two studies [14, 15]. The latter also reported a significantly higher LVEDS. Binnetoglu *et al*. [16] reported similar mean values for LVEDD and LVESD in athletes and controls, except for basketball players. Athletes’ IVS was significantly higher in three studies [14, 15, 16], LVPWT was significantly increased in five studies [14, 15, 16, 17, 18]. Sulovic *et al*. [18] reported a higher RWT in athletes. In addition, static exercising athletes had a significantly higher RWT compared to dynamic exercising athletes. The same for LVM held true in this study and in three other studies [14, 15, 17, 18]. Binnetoglu *et al*. [16] reported the same findings in soccer players vs. tennis players and controls and a significantly increased LVM in wrestlers vs. tennis players. In summary, there is evidence of structural cardiac adaptations in junior athletes. These adaptations cannot be exclusively attributed to either dynamic or static types of sport (see **Supplementary Table 6**).

#### 3.6.2 Left Ventricular Function

LV systolic function is represented by stroke volume (SV), LV ejection fraction (EF), fractional shortening (FS), cardiac output (Q), cardiac index, peak systolic myocardial velocity (S’), LV myocardial performance index (Tei index), concentricity, and sphericity index.

Endurance athlete’s EF was significantly increased in the study by Rundqvist *et al*. [14]. Divided into dynamic and static types of sport, Sulovic *et al*. [18] reported a significantly higher EF in dynamic exercising athletes and a significantly reduced EF in static exercising athletes. Contrary to these findings, three studies did not report significant differences [15, 16, 17]. The same is reported for FS [15, 16].

Diastolic function is represented by eleven parameters: mitral annulus plane systolic excursion (MAPSE), peak early LV diastolic filling velocity (E), peak late LV diastolic filling velocity (A), E/A ratio (E/A), deceleration time of E (DT), early diastolic myocardial velocity (E’), late diastolic myocardial velocity (A’), E/E’ ratio (E/E’), and E’/A’ ratio (E’/A’).

Significant differences for E and A are reported by Binnetoglu *et al*. [16] and Sulovic *et al*. [18]. Swimmers’ E was significantly higher than the other athletes and the CG [16]. Sulovic *et al*. [18] reported a significantly higher E in dynamic compared to static exercising athletes, and a significantly reduced E in the latter, compared to controls. Wrestlers [16] had a reduced A compared to controls as well as static exercising athletes compared to dynamic exercising athletes and controls [18]. E/A was significantly higher in athletes in the study by Rundvqist *et al*. [14]. Summarized, the studies reported contradictory findings regarding LV function (**Supplementary Table 7**).

#### 3.6.3 Right Ventricular Structure

Two studies assessed RV structure [3, 14] with the following parameters: right ventricular outflow tract (RVOT) assessed in the parasternal long-axis view (PLAX) and the parasternal short-axis view (PSAX), RVOT distal diameter, RV basal diameter, RV mid-cavity diameter, RV end-diastolic area, and RV end-systolic area. All RV parameters in D’Ascenzi *et al*.’s [3] study failed significance (indexed to BSA) except for RV end-systolic area. RV parameters by Rundqvist *et al*. [14] were significantly higher in athletes compared to controls (*p <* 0.01). In summary, there are conflicting results on the effect of exercise on RV structure (**Supplementary Table 8**).

#### 3.6.4 Right Ventricular Function

Diastolic parameters as tricuspid annular plane systolic excursion (TAPSE), E/A, E’, A’, E/E’, and E’/A’ were assessed by two authors [3, 14]. RV systolic function was assessed with two parameters: S’ and RV fractional area change (FAC). Only TAPSE, indexed to BSA, was significantly higher in athletes whereas RV FAC was significantly reduced [14]. Summarized, there are conflicting results on the effect of exercise on RV function (**Supplementary Table 9**).

#### 3.6.5 Left Atrial Structure

D’Ascenzi *et al*. [13] reported results of biatrial remodelling. Four other authors [14, 15, 17, 18] assessed LA diameter and LA volume. There were no significant differences in the study by D’Ascenzi *et al*. [13] and Sulovic *et al*. [18]. Rundqvist *et al*.’s [14] study showed an increased LA diameter and volume. Soccer players in the study by Zdravkovic *et al*. [15] had an increased diameter compared to controls. Summarized, three out of five studies reported increased left atria dimensions in athletes (**Supplementary Table 10**).

#### 3.6.6 Right Atrial Structure

Two authors reported results of RA structure [13, 14]. Parameters assessed were: RA area, RA diameter, and RA volume. Soccer players in the study by D’Ascenzi *et al*. [13] had a significantly larger RA volume compared to controls. Rundqvist *et al*. [14] observed a significantly increased RA area and diameter in endurance athletes. Summarized, there is evidence of the influence of exercise on right atrial structure (**Supplementary Table 11**).

### 3.7 2D Speckle Tracking Echocardiography 

Six of the eight studies assessed myocardial strain by 2D speckle tracking echocardiography [3, 12, 13, 14, 16, 17]. Four studies assessed LV function [12, 14, 16, 17], two authors focused on the RV [3, 14], and/or function of the atria, respectively [13, 14]. This categorization was further followed to compare studies’ results. All studies applied the same software for off-line analysis (EchoPAC, GE Healthcare), but used different versions. All performed the analysis from 40 frames/s to 80–100 frames/s and measured myocardial movement selecting the heart cycle with the most defined endocardial border at end-diastole. Authors, however, applied different recommendations on how to perform 2D STE (**Supplementary Table 4**).

#### 3.7.1 Left Ventricular Function

Three studies [12, 16, 17] reported results on four-chamber longitudinal strain. Whereas Beaumont *et al*. [12] did not observe significant differences between soccer players and controls, basketball players in the study by Binnetoglu *et al*. [16] had a significantly lower strain (*p <*0.0001) compared to soccer players, swimmers, wrestlers, and controls but not tennis players. Controls in the study by Simsek *et al*. [17] had a significantly lower strain compared to runners and wrestlers. The authors observed the same for the two- and three-chamber view as well, with a significantly lower strain in controls. The global longitudinal strain (GLS) as an overall marker of LV function was assessed in three studies [14, 16, 17]. Binnetoglu *et al*. and Rundqvist *et al*. [14, 16] reported GLS as an average strain of 18 segments (four-, two-, and three-chamber view, two walls each, subdivided into basal, mid, and apical segments). Simsek *et al*. [17] however, reported GLS as an average strain of 15 segments.

Again, basketball players in the study by Binnetoglu *et al*. [16] presented the lowest GLS compared to other study groups (*p <* 0.001). Simsek *et al*. [17] did not observe a significant difference in GLS between runners and wrestlers but a significant difference between the two athlete groups and controls was reported. Rundqvist *et al*. [14] on the contrary did not observe a significant difference in GLS between endurance athletes and controls.

Two studies [12, 16] reported results on circumferential and radial strain but for different LV segments. Beaumont *et al*. [12] measured circumferential and radial strain at the mitral valve or basal level, respectively, and mid-ventricular at the mid-papillary muscle level [12]. Circumferential strain differed significantly at both levels between soccer players and controls with higher values in soccer players. They did not observe significant differences regarding radial strain. In contrast, Binnetoglu *et al*. [16] reported global circumferential and radial strain, measured at the anteroseptal, anterior, lateral, posterior, inferior, and septal wall but did not state at which segmental level [16]. The combined group of athletes showed a significantly lower circumferential strain (*p <* 0.04), however, post-hoc analysis did not reveal significant differences between groups. Only Beaumont *et al*. [12] assessed rotational and twist mechanics and found a significant difference between soccer players and controls in counter-clockwise apical rotation. In summary, three out of four studies reported higher strain values in athletes with results being influenced by the type of sports (**Supplementary Table 12**).

#### 3.7.2 Right Ventricular Function

D’Ascenzi *et al*. [3] and Rundqvist *et al*. [14] reported RV longitudinal strain values assessed at the four-chamber view. Both authors took measurements of the RV free wall only, subdivided into basal, mid, and apical segments, and did not observe significant differences between athletes and controls (*p >* 0.05), see **Supplementary Table 13**.

#### 3.7.3 Left Atrial Function

Two of six studies reported results on 2D STE of the LA, however, D’Ascenzi *et al*. [13] and Rundqvist *et al*. [14] did not investigate the same LA parameters. D’Ascenzi *et al*. [13] reported results on peak atrial longitudinal strain (PALS), which is a measure of LA deformation during the reservoir phase, and peak atrial contraction strain (PACS), which is the myocardial strain during atrial systole [21, 22]. They did not report significant differences between athletes and controls. Rundqvist *et al*. [14] assessed LA total strain measured at the four- and two-chamber view with subdividing the LA into six segments each [23] and also did not observe significant differences between athletes and controls either. Summarized, there is no evidence of an influence of exercise on LA strain, (**Supplementary Table 13**).

#### 3.7.4 Right Atrial Function

Only D’Ascenzi *et al*. [13] reported results on 2D STE of the right atrium, assessed at the four-chamber view with subdividing RA into six segments. Analogous to LA function, PALS and PACS of the right atrium were assessed. The authors did not find significant differences between athletes and controls. Summarized, there is no evidence for an influence of exercise on RA strain (**Supplementary Table 13**).

## 4. Discussion

This systematic review compared results of eight studies assessing 2D TTE and 2D STE parameters in junior athletes vs. an inactive CG. The main findings of the study were: (1) Training-induced chamber-remodelling does occur in junior athletes. (2) Results regarding 2D TTE assessed LV and RV function are conflicting and do not provide a clear statement pointing towards an improved function in athletes. (3) LV function assessed by 2D STE was improved in junior athletes in two of three studies. RV and atrial function were not affected by exercise.

### 4.1 2D Transthoracic Echocardiography

#### 4.1.1 Left Ventricular Structure

Overall, five of six studies observed increased LV dimensions. These results are in line with other authors [4, 5, 7, 8, 24, 25, 26]. Mc Clean *et al*. [4], reported increased LV morphometry in a meta-analysis involving >14 000 junior athletes. LVEDD, LVDS, IVSD, RWT, LVM (*p *≤ 0.001), and LVPWT (*p *≤ 0.01) differed by 5.6–27.6% from non-athletic controls. Krysztofiak *et al*. [25] observed a significantly increased LVEDD, LVPWT (*p <* 0.001) and IVS (*p <* 0.01) in 791 boys and girls of different types of sports (5–18 years) and Sharma *et al*. [7] significantly higher IVSD, LVPWT, LVWT, LVEDD. Non-significant LV structure in soccer players compared to controls by Beaumont *et al*. [12] were explained by participants maturity status, referring to Nottin *et al*. [27] who concluded that a sufficient maturity status has to be reached to elicit an exercise-induced increase in LV structure. This observation is not in line with results by Mc Clean *et al*. [4] who observed a significant influence of exercise on cardiac dimensions during as well as before puberty. Ayabakan *et al*. [5], reported a significantly increased IVSD, LVPWT, LVMI (*p <* 0.001), and RWT (*p <* 0.007) in 22 pre-pubertal male swimmers (10–12 years). These results, as well as results by D’Ascenzi *et al*. [3], interfere with the hypothesis by Nottin *et al*. [27], leading to the assumption that adaptations do take place before and during puberty – however adaptations might be accelerated by hormonal influences during puberty. Additionally, other factors contribute to cardiac adaptations, such as genetics, training history and intensity.

LV hypertrophy, defined by an LVM >95th percentile can be differentiated into eccentric (RWT ≤0.42) and concentric (RWT >0.42) hypertrophy [28]. Traditionally, it is believed that dynamic stimuli result in eccentric and static stimuli in concentric hypertrophy, respectively [29, 30]. Binnetoglu *et al*. [16] observed LV hypertrophy in 45.9% of all athletes with 29% being eccentric and 16.1% concentric. Interestingly, the type of LV hypertrophy was not a function of the underlying training stimulus. More than one third (35.6%) of swimmers (static-dynamic) as well as 39.1% of wrestlers (static) presented eccentric hypertrophy whereas concentric hypertrophy was observed in 28.9% of swimmers and only 4.3% of wrestlers. Sulovic *et al*. [18] reported eccentric hypertrophy in 79.4% of athletes in the dynamic group and 54.05% of the static group. Concentric hypertrophy was prevalent in 20.6% of athletes of the dynamic and 49.95% of the static groups. On the contrary, Simsek *et al*. [17] reported results that support the traditional view. Participants in this study [17] were slightly older compared to Binnetolgu *et al*. [16] and Sulovic *et al*. [18]. Aformentioned influences like hormonal status, genetics and training history could play a role in this adaptive process.

Regarding LV wall dimensions, a LVWT or IVSD >12 mm represents the upper limit in males, and >11 mm the upper limit in females [7]. Binnetoglu *et al*. [16] reported 98.9% of male athletes having IVSD >12 mm. Other authors [14, 15, 17, 18] reported none of the athletes exceeding this cut-off which is in line with Pelliccia *et al*. [6] who reported LVWT >12 mm in only 16 of 947 male elite Olympic athletes (13–49 years) as well as results by Sharma *et al*. [7] who reported LVWT >12 mm in 38 out of 720 male elite athletes (15.7 ± 1.4 years). None of the studies included in this review reported IVSD or LVWT >11 mm for females. During puberty, testosterone levels in males exceed female levels by up to 15 times [31]. That is why structural changes in males may be more pronounced than in females.

In conclusion, exercise does have an impact on LV structure in young athletes. This impact is influenced by athletes’ age, hence pubertal and hormonal status and also by training volume and intensity. Most studies observed significantly increased LV diameter, LVWT, and LVM in athletes.

#### 4.1.2 Left Ventricular Function

Results regarding LV function did not clearly state significantly different results between athletes and controls or within the athletic groups. None of the studies reported any adverse results regarding a significantly impaired LV function. Three authors [12, 14, 18] noticed an improved systolic function by a significantly increased EF in soccer players and dynamic sports, respectively. Rundqvist *et al*. [14] observed a significantly improved diastolic function (E/A) as well as Sulovic *et al*. [18] in endurance-trained athletes (E). This result is in line with other studies [5, 24, 32, 33]. Unnithan *et al*. [33] compared 22 highly trained soccer players (12 ± 0.3 years) and 15 controls (11.7 ± 0.2 years) and noticed a significantly higher E in soccer players at rest as well as during submaximal exercise on the cycle ergometer. The authors concluded that exercise leads to an improved diastolic function in highly trained athletes even at a young age. Ayabakan *et al*. [5] and Rundqvist *et al*. [34] noticed significantly improved diastolic function in pre-pubertal male swimmers (10–12 years) and endurance athletes (13–19 years), respectively. Gajda *et al*. [35] examined 12 swimmers at a ultramarathon-relay with TTE before, during the competition and during recovery, e.g., 48 hours after the competition. During recovery, LV EF and SF were significantly increased compared to baseline measurements and during the competition. On the contrary, Pavlik *et al*. [32] compared male children, adolescents, and adults with a significantly improved diastolic function in adolescents (15–18 years) and adults (19–60 years), only. McClean *et al*. [4] and Sharma *et al*. [7] did not report a significantly improved diastolic function at all, and Sulovic *et al*. [18] noticed a reduced diastolic function compared to controls (E, A) in static training athletes.

In conclusion, results are controversial and do not allow a clear statement. Regarding LV systolic function, there are studies reporting improved results in young athletes but also no significantly different results compared with controls. The same is for LV diastolic function. If significantly increased results were reported, they were reported in endurance athletes but not in strength-trained athletes.

#### 4.1.3 Right Ventricular Structure

Two studies assessed RV structure [3, 14] with conflicting results. D’Ascenzi *et al*. [3] only noted a significant increase in RV end-systolic area index in swimmers compared to controls. All other parameters failed statistical significance when indexed to BSA. Rundqvist *et al*. [14] observed a significantly increased RVOT, RV basal diameter index, and RV end-systolic area index. RV adaptation is expected in athletes as the RV works hand-in-hand with the LV [36, 37]. Strength training, on the contrary, does not affect the RV to the same extent that endurance exercise does, and pulmonary vasculature is protected by high pressures [30, 38]. Current literature does not provide better insight into RV structure in children and adolescents. Only one study could be found that assessed RV structure in this age group [39]. Allen *et al*. [39] reported RVWT and RV cavity in 77 swimmers (32 females), aged 10.8 (5–17) years. All participants exceeded the 95th percentile of reference values for RVWT, and most of the participants for RV cavity. La Gerche *et al*. [38] noticed a significant increase in RV volume right after a competition in 40 adult athletes (37 ± 8 years) and hypothesize a strong impact of endurance exercise on the right heart’s structure. Comparisons of RV end-diastolic and end-systolic areas were significantly higher in adult athletes vs. controls [40] and endurance athletes vs. strength-trained athletes [41]. In conclusion, results are controversial. Results in adults point towards an influence of predominantly dynamic but not strength exercise. Further studies are needed to confirm these results in the younger age group.

#### 4.1.4 Right Ventricular Function

Two studies investigated RV function in junior athletes [13, 14]. Rundqvist *et al*. [14] found a functional remodelling in endurance-trained athletes whereas D’Ascenzi *et al*. [3] observed no differences between swimmers and controls for most parameters and a significantly reduced RV FAC in swimmers. La Gerche *et al*. [38, 42] confirmed these results in adults at rest and immediately after a competition. Thus, the slightly reduced resting function that was preserved during exercise rather bears a contractile reserve but does not represent impaired RV function [42]. Reduced RV function in highly trained athletes immediately after a competition mostly recovered after one week but long-term structural remodelling is likely [38]. The adverse consequence of this is ventricular arrhythmia, which is observed in trained adults, associated with a longer duration of exercise [29, 38, 43]. To prevent this adverse adaptation in children and adolescents, a closer observation of junior athletes is needed—especially in a longitudinal setting.

#### 4.1.5 Left and Right Atrial Structure 

Five studies examined LA structure [13, 14, 15, 17, 18], and two studies assessed RA structure [13, 14]. All studies except for two [13, 18] reported significantly increased LA and RA dimension and volumes which is in line with studies in adult athletes [44, 45, 46, 47, 48, 49]. During exercise, the LA adapts to pressure and volume overload, which leads to LA dilatation [45]. Pelliccia *et al*. [48] observed marked atrial dilatation (≥40 mm) in 20% of n = 1777 adult athletes but defined this as a physiological adaptation to exercise as only 0.8% of athletes presented with supraventricular arrhythmias. LA adaptation was largely associated with the LV as 1 mm in LV dilatation induced a 0.4 mm increase in LA diameter. Furthermore, LVWT, BSA, and age contributed to LA adaptation. For the RA, increased diameter and volume were also regarded as a physiological adaptation [13, 49]. Gjerdalen [47], however, noticed significantly more tricuspid regurgitations in adult athletes vs. controls (n = 343/58% vs. 17/36%).

In conclusion, LA diameter and volume, RA volume, area, and diameter were increased in athletes, indicating a significant influence of exercise. As a consequence of LA dilatation, atrial flutter or fibrillation could arise as complication. Therefore, more focus should be placed on atrial examination to detect adverse adaptations as early as possible.

### 4.2 2D Speckle Tracking Echocardiography

Six of eight studies included in this review performed STE analysis [3, 12, 13, 14, 16, 17]. 2D STE is accepted as an early marker for systolic dysfunction as it detects a decrease in contractility when EF is still within normal limits [50, 51].

#### 4.2.1 Left Ventricular Function

Two of four studies reported improved LV function in junior athletes. In the study by Simsek *et al*. [17], this difference did not depend on the types of sports, as there were no significant differences between endurance and strength athletes. On the contrary, Binnetoglu *et al*. [16] observed a significantly reduced strain in basketball players compared to other groups and controls whereas Rundqvist *et al*. [14] did not observe significant differences in strain between endurance athletes and controls. The latter is in line with no significant results reported by other authors [33, 52, 53] who compared 22 soccer players (12.0 ± 0.3 years) with 15 controls (11.7 ± 0.2 years) at rest and during submaximal exercise [33], and 76 cross country skiers (12.1 ± 0.2 years) with 25 controls (12.1 ± 0.3 years) [52]. No significant differences for GCS and GRS were reported by Charfeddine *et al*. [54] in a cohort of 33 soccer players (13.19 ± 1.2 years) and 20 controls (12.9 ± 2.1 years). Furthermore, GLS turned out to be significantly reduced in athletes (20.68 ± 2.05 vs. 22.99 ± 2.32, *p <* 0.001). De Luca *et al*. [55] support these results. They recognized reduction in LV strain as an early sign of LV dysfunction in n = 50 athletes (soccer, cycling, basketball, 14–19 years). In conclusion, results are conflicting regarding LV function. Contrary to the majority of studies [14, 33, 52, 53, 54], two studies presented in this review [12, 17] reported an improved LV function in junior athletes.

#### 4.2.2 Right Ventricular Function

Two studies assessed RV function in junior athletes [3, 14] and did not observe significantly different values in athletes and controls. Furthermore, there was a tendency toward lower strain values in athletes vs. controls. Bjerring *et al*. [52] observed a significantly reduced RV GLS (28 ± 4 vs. 31 ± 3, *p <* 0.001) in 76 cross country skiers (12.1 ± 0.2 years) compared to 25 controls (12.1 ± 0.3 years) and a negative correlation of RV GLS with ${\mathrm{VO}}_{2\,m\,a\,x}$ (r = –0.22, *p <* 0.06) and the amount of exercise (r = –0.24, *p <* 0.05). They hypothesize that the RV might be affected by cardiac fatigue through exercise which is explained by a reduced resting cardiac function in athletes after strenuous exercise. This cardiac fatigue might affect the RV earlier than the LV [38, 52]. In conclusion, results of this review do not point towards a significant influence of exercise on RV longitudinal strain. However, only two studies out of six reported RV longitudinal strain. There is evidence that the RV is affected by strenuous exercise. As the RV bears the potential to elicit arrhythmogenic cardiomyopathies, the focus of further studies should be placed of the assessment of RV function.

#### 4.2.3 Left and Right Atrial Function

Two studies investigated atrial function by 2D STE [13, 14]. D’Ascenzi *et al*. [13] examined biatrial function in swimmers vs. controls and did not report significant differences. Rundqvist *et al*. [14] observed non-significant differences in LA strain between endurance athletes and controls. One study was identified that examined LA function in n = 595 highly trained soccer players (25.1 ± 4.6 years) and n = 47 controls (26.2 ± 6.5 years) with no significant differences between groups [47]. Furthermore, in athletes with enlarged atria, LA function was still preserved. In conclusion, results do not point towards a significant influence of exercise on LA strain parameters. However, only two studies of six investigated LA strain. Only one study examined RA function and did not observe a significant influence of exercise. As both atria, are affected by higher blood volume in athletes, and the LA also by an increase in pressure during exercise, assessing atrial function is of importance.

### 4.3 Limitations

The number of studies investigating cardiac adaptations in young athletes is limited. Comparability of existing studies is difficult due to differences in age groups, different types of sports, whether male or female athletes are being compared, and, importantly the difference in the parameters assessed by echocardiography itself. The latter calls for a consensual recommendation on how to assess the pediatric athlete’s heart by 2D TTE and 2D STE and on how to report these data [56]. If available, sex- and age-dependent z-scores should be reported instead of absolute values [4, 56]. In total, 51 parameters have been assessed by 2D TTE in eight studies, regarding the left and right heart structure and function, and 15 different parameters with 2D STE. All parameters have been assessed in different cohorts, with sub-groups of n = 16 to n = 100 participants, predominantly males. The age varied from 10.8 ± 0.2 to 17.5 ± 2.2 years, including pre-, peri-, and post-pubertal athletes, respectively. This variation in-between and within studies itself complicates the comparison of echocardiographic parameters, that should be discussed regarding age and pubertal status, respectively. Additionally, sex, ethnicity, and genetic influences contribute to the variability in results [4, 29, 31, 37, 57, 58]. Regarding sex, females are not represented equally in the literature. Out of >14 000 young athletes in the review by McClean *et al*. [4], only 19% of participants were females. Studies included in this review include Caucasian athletes only. In general, cardiac adaptations are more pronounced in athletes of African ethnicity compared to other ethnicities [59, 60, 61]. It is not possible to rule these influences out, especially the role of genetics. Therefore, considerable care should be taken to ensure balanced study groups. In this review, participants took part in 10 different types of sport. As the training stimulus is known to influence physical adaptation, this is a further source of controversial results. Genetic traits, determine the type of sports a child chooses to some extent [33], depending on the ability to perform successfully in the chosen discipline. By comparing adequate samples of different types of sports, this impact could be accounted for. The definition of inactive controls varied considerably within the studies. Authors defined their control groups as either being sedentary [16, 17], not engaged in regular exercise or competitive sports [14, 18] or not exercising more than 2 hours/week [3, 13, 15]. Only one study [12] specified the activity of the control group with 1.53 ± 1.77 hour of recreational physical activity/week. Fagard *et al*. [62] observed cardiac adaptations to happen if subjects exercise ≥3 hours/week. This activity level of the control group could have led to non-significant differences between athletes and controls in this study.

Most echocardiographic parameters are indexed to BSA, to account for anthropometric differences in subjects and to enable comparability [19]. However, how parameters were indexed contributed to variation in echo parameters. Diameters were either not indexed to BSA or indexed to BSA or ${\mathrm{BSA}}^{0.5}$, volumes were either not indexed to BSA or indexed to BSA or ${\mathrm{BSA}}^{1.5}$. LVM can be indexed to BSA, ${\mathrm{BSA}}^{1.5,}$ or body ${\mathrm{height}}^{2.7}$ ($m^{2.7}$). Additionally, three different methods to calculate BSA are reported in the studies presented: the Mosteller method [63], the Du Bois formula [64], and the Haycock formula [65]. The Mosteller formula was proven to be a reliable estimate of BSA in children [66]. There are studies reporting a good correlation between the Mosteller and Du Bois as well as between the Mosteller and Haycock formula (r = 0.99 for both) [67] however, some studies revealed considerable differences [68, 69]. Guidelines, providing a concept on which formula to apply, and on how to report indexed results are needed to overcome this large variability in data.

Overall, as requested by D’Ascenzi [44], recommendations on how to assess cardiac function in this pediatric sub-group, are required. In addition, studies that assess cardiac function in a longitudinal setting [33] could provide better insight into the process of cardiac adaptation in junior athletes, help us to differentiate between physiological and pathological adaptations and to recognize these differences at a very early stage.

## 5. Conclusions

Cardiac adaptation to exercise does occur in children and adolescents—even in very young athletes. These adaptations are more pronounced in structural parameters, whereas functional parameters are preserved or slightly improved. The underlying stimuli for cardiac adaptation have been identified as being factors like the training history, training volume and intensity, the types of sports [70], genetics [58] and pubertal and hormonal status [31, 71, 72].

The variability, given by the nature of the cohort of junior athletes and the individual sports emphasizes the need to standardize variables, e.g., the test and measures we apply and how results are reported. Recommendations on the assessment of cardiac function in junior athletes are needed as well as studies with a longitudinal design.

## Abbreviations

2D STE, 2-dimensional speckle tracking echocardiography; 2D TTE, 2-dimensional transthoracic echocardiography; A, peak late LV diastolic filling velocity; A’, late diastolic myocardial velocity; BMI, body mass index; BSA, body surface area; CG, control group; DBP, diastolic blood pressure; DT, deceleration time of E; E, peak early LV diastolic filling velocity; E’, early diastolic myocardial velocity; E’/A’, E’/A’ ratio; E/A, E/A ratio; E/E’, E/E’ ratio; EF, ejection fraction; FAC, fractional area change; FS, fractional shortening; GLS, global longitudinal strain; IVS, interventricular septal thickness; LV, left ventricle; LVEDD, LV end-diastolic diameter; LVEDV, LV end-diastolic volume; LVESD, LV end-systolic diameter; LVESV, LV end-systolic volume; LVM, left ventricular mass; LVPWT, LV posterior wall thickness; LVWT, LV wall thickness; MAPSE, mitral annulus plane systolic excursion; MWT, mean wall thickness; PACS, peak atrial contraction strain; PALS, peak atrial longitudinal strain; PLAX, parasternal long-axis view; PSAX, parasternal short-axis view; Q, cardiac output; RVOT, right ventricular outflow tract; RWT, relative wall thickness; S’, peak systolic myocardial velocity; SBP, systolic blood pressure; SV, stroke volume; TAPSE, tricuspid annular plane systolic excursion.

## Supplementary Material

Supplementary material associated with this article can be found, in the online version, at https://doi.org/10.31083/j.rcm2304129.
