# Supplementary material for: Cardiac Structure and Function in Junior Athletes: A Systematic Review of Echocardiographic Studies
Source: Rev Cardiovasc Med. 2022 Apr 7;23(4):129. doi: 10.31083/j.rcm2304129 (PMC11273646; doi:10.31083/j.rcm2304129)
Supplement: Supplementary file 1 [file 2153-8174-23-4-129-s1.docx]

**Supplementary Table 1. Risk of bias assessment for methods.**

| **Risk of bias assessment: methods** | | **Beaumont et al.  (2019)** | **Binnetolgu et al.  (2017)** | **D'Ascenzi et al.  (2017)** | **D'Ascenzi et al.  (2016)** | **Rundqvist et al.  (2016)** | **Simsek et al.  (2013)** | **Sulovic et al.  (2017)** | **Zdravkovic et al.  (2010)** | **Summary** |
| --- | --- | --- | --- | --- | --- | --- | --- | --- | --- | --- |
|  | **Criterion** |  |  |  |  |  |  |  |  |  |
| **Selection bias** | Were cases and controls selected appropriately (e.g., appropriate diagnostic criteria  or definitions, equal application of exclusion criteria to case and controls, sampling  not influenced by exposure status) | **x** | **x** | **x** | **x** | **x** | **x** |  | **x** | **87.5%** |
|  | Does the design or analysis control account for important confounding and modifying  variables through matching, stratification, multivariable analysis, or other approaches? |  |  | **x** | **x** |  |  |  | **x** | **37.5%** |
| **Performance bias** | Did researchers rule out any impact from a concurrent intervention or an unintended  exposure that might bias results? |  |  |  |  |  |  |  |  | **0%** |
|  | Did the study maintain fidelity to the intervention protocol? | **x** | **x** | **x** | **x** | **x** | **x** | **x** | **x** | **100%** |
| **Attrition bias** | If attrition (overall or differential nonresponse, dropout, loss to follow-up, or exclusion  of participants) was a concern, were missing data handled appropriately  (e.g., intention-to-treat analysis and imputation)? |  |  |  |  |  |  |  |  | **0%** |
| **Detection bias** | In prospective studies, was the length of follow-up different between the groups, or in  case-control studies, was the time period between the intervention/exposure and outcome the same for cases and controls? | **x** | **x** | **x** | **x** | **x** | **x** | **x** | **x** | **100%** |
|  | Were the outcome assessors blinded to the intervention or exposure status of participants? |  |  |  |  |  |  |  | **x** | **12.5%** |
|  | Were interventions/exposures assessed/defined using valid and reliable measures, implemented consistently across all study participants? | **x** | **x** | **x** | **x** | **x** | **x** | **x** | **x** | **100%** |
|  | Were outcomes assessed/defined using valid and reliable measures, implemented  consistently across all study participants? | **x** | **x** | **x** | **x** | **x** | **x** | **x** | **x** | **100%** |
|  | Were confounding variables assessed using valid and reliable measures, implemented  consistently across all study participants? | **x** | **x** | **x** | **x** |  |  | **x** | **x** | **75%** |
| **Reporting bias** | Were the potential outcomes prespecified by the researchers? Are all prespecified outcomes reported? | **x** | **x** | **x** | **x** | **x** | **x** | **x** | **x** | **100%** |

| **Supplementary Table 2. Risk of bias assessment for methods.**  **Risk of bias assessment: results** | | | | | | | | | | | | | |  |
| --- | --- | --- | --- | --- | --- | --- | --- | --- | --- | --- | --- | --- | --- | --- |
|  | **Criterion 1** | **Criterion 2** | **Criterion 3** | **Criterion 4** | **Criterion 5** | **Criterion 6** | **Criterion 7** | **Criterion 8** | **Criterion 9** | **Criterion 10** | **Criterion 11** | **Criterion 12** |  |  |
|  |  |  |  |  |  |  |  |  |  |  |  |  |  |  |
|  |  |  |  |  |  |  |  |  |  |  |  |  |  |  |
|  | **Athletes details specified** | **Assessment of athletes fitness level (VO_2max_)** | **Non-active controls^a^** | **Age-matched  controls** | **Puberty status** | **Non-significant anthropometric differences in athletes vs. controls** | **Non-significant physiologic differences^b^ in athletes vs. controls** | **Measurements performed according to standardized guidelines** | **One observer performing all measurements/ Interobserver reliability** | **Statistical methods explained in detail** | **No missing data, all  p-values given** | **No inconsistent/ incorrect data^c^** | **Total (%)** |  |
| **Author** |  |  |  |  |  |  |  |  |  |  |  |  |  |  |
| **Beaumont et al. 2019** | **x** |  | **x** |  | **x** | **x** | **x** | **x** |  | **x** | **x** | **x** | 9 (75%) |  |
| **Binnetoglu et al. 2017** | **x** |  | **x** |  |  |  |  | **x** |  | **x** | **x** | **x** | 6 (50%) |  |
| **D'Ascenzi et al. 2017** | **x** |  | **x** | **x** | **x** | **x** |  | **x** | **x** | **x** | **x** | **x** | 10 (84%) |  |
| **D'Ascenzi et al. 2016** | **x** |  | **x** | **x** | **x** | **x** |  | **x** | **x** | **x** | **x** | **x** | 10 (84%) |  |
| **Rundqvist et al. 2016** | **x** | **x** | **x** | **x** |  | **x** | **x** | **x** | **x** | **x** | **x** | **x** | 11 (92%) |  |
| **Simsek et al. 2013** | **x** |  | **x** | **x** |  | **x** | **x** | **x** | **x** | **x** | **x** | **x** | 10 (84%) |  |
| **Sulovic et al. 2017** | **x** |  | **x** | **x** |  |  |  | **x** |  | **x** | **x** | **x** | 7 (59%) |  |
| **Zdravkovic et al. 2010** | **x** |  | **x** | **x** |  |  |  | **x** | **x** | **x** | **x** | **x** | 8 (67%) |  |
|  |  |  |  |  |  |  |  |  |  |  |  |  |  |  |
|  |  |  |  |  |  |  |  |  |  |  |  |  |  |  |
| ^a^ Non-active controls are defined by not participating in organized physical activity/ exercise training of > 3 h/week. | | | | | | | | | | | | | |  |
| ^b^ Physiologic differences reffering to blood pressure. | | | | | | | | | | | | | |  |
| ^c^ For any data in question, the authors were contacted and asked for clarification. If there was no answer or the data in quesiton could not be clarified, 0 P were assigned. If data could be clarified, x=1 P was assigned. | | | | | | | | | | | | | |  |

.

**Supplementary Table 3. Study quality assessment.**

|  | **Beaumont 2019** | | | **Binnetoglu 2017** | | | **D'Ascenzi 2017** | | | **D'Ascenzi 2016** | | | **Rundqvist 2016** | | | **Simsek 2013** | | | | **Sulovic 2017** | | | **Zdravkovic 2010** | | |  |  |  |
| --- | --- | --- | --- | --- | --- | --- | --- | --- | --- | --- | --- | --- | --- | --- | --- | --- | --- | --- | --- | --- | --- | --- | --- | --- | --- | --- | --- | --- |
|  | Y | N | X | Y | N | X | Y | N | X | Y | N | X | Y | N | X | Y | N | X | Y | | N | X | Y | N | X |  |  |  |
|  |  |  |  |  |  |  |  |  |  |  |  |  |  |  |  |  |  |  |  |  |  |  |  |  |  |  |  |  |
| Was the research question or objective in this paper clearly stated and appropriate? | x |  |  | x |  |  | x |  |  | x |  |  | x |  |  | x |  |  | x | |  |  | x |  |  |  |  |  |
| Was the study population clearly specified and defined? | x |  |  | x |  |  | x |  |  | x |  |  | x |  |  | x |  |  | x | |  |  | x |  |  |  |  |  |
| Did the authors include a sample size justification? |  | x |  |  | x |  |  | x |  |  | x |  |  | x |  |  | x |  |  | | x |  | x |  |  |  |  |  |
| Were controls selected or recruited from the same or similar population that gave rise to the cases (including the same timeframe)? | x |  |  |  |  | NR | x |  |  | x |  |  | x |  | NR |  |  | NR |  | |  | NR |  |  | NR |  |  |  |
| Were the definitions, inclusion and exclusion criteria, algorithms or processes used to identify or select cases and controls valid, reliable, and implemented consistently across all study participants? | x |  |  | x |  |  | x |  |  | x |  |  | x |  |  |  | x |  |  | | x |  | x |  |  |  |  |  |
| Were the cases clearly defined and differentiated from controls? | x |  |  | x |  |  | x |  |  | x |  |  | x |  |  | x |  |  | x | |  |  | x |  |  |  |  |  |
| If less than 100 percent of eligible cases and/or controls were selected for the study, were the cases and/or controls randomly selected from those eligible? | x |  |  |  |  | NA | x |  |  | x |  |  |  |  | NA |  |  | NA |  | |  | NA |  |  | NA |  |  |  |
| Was there use of concurrent controls? | x |  |  |  |  | NR |  |  | NR |  |  | NR |  |  | NR |  |  | NR |  | |  | NR |  |  | NR |  |  |  |
| Were the investigators able to confirm that the exposure/risk occurred prior to the development of the condition or event that defined a participant as a case? | x |  |  | x |  |  | x |  |  | x |  |  | x |  |  | x |  |  | x | |  |  | x |  |  |  |  |  |
| Were the measures of exposure/risk clearly defined, valid, reliable, and implemented  consistently (including the same time period) across all study participants? | x |  |  | x |  |  | x |  |  | x |  |  | x |  |  | x |  |  | x | |  |  | x |  |  |  |  |  |
| Were the assessors of exposure/risk blinded to the case or control status of participants? |  |  | NR |  |  | NR |  | x |  |  | x |  |  |  | NR |  | x |  |  | |  | NR | x |  |  |  |  |  |
| Were key potential confounding variables measured and adjusted statistically in  the analyses? If matching was used, did the investigators account for matching during  study analysis? |  | x |  |  | x |  | x |  |  | x |  |  | x |  |  |  | x |  |  | | x |  |  | x |  |  |  |  |
|  |  |  |  |  |  |  |  |  |  |  |  |  |  |  |  |  |  |  |  | |  |  |  |  |  |  |  |  |
| Quality Rating (Good, Fair, or Poor) | GOOD | | | FAIR | | | GOOD | | | GOOD | | | GOOD | | | POOR^a^ | | | | POOR^b^ | | | GOOD | | |  | |  |
| * CD - cannot determine. NA - not applicable. NR - not reported.  ^a^ No evaluation of physical activity behaviour in controls; no sample size calculation; weak statistics, no covariance analysis.  ^b^ Insufficient description of recruitment of controls; no evaluation of physical activity behaviour in athletes and controls; no application of inclusion criteria for controls; no sample size calculation weak statistics, no   covariance analysis. | | | | | | | | | | | | | | | | | | | | | | | | | | | | |

|  |  |  |  |  |  |  |  |  |  |  |  |  |  |
| --- | --- | --- | --- | --- | --- | --- | --- | --- | --- | --- | --- | --- | --- |

**Supplementary Table 4. Study charcteristics.**

| **Author** | **Athletes** | **Mitchell Classification** | **4-Step-Classification** | **Sex  [m/f]** | **Age  [years]** | | **Athletes' training profile** | **Controls** | **Sex  [m/f]** | **Age  [years]** | | **Controls' profile** | | **Pubertal status** | | **2D Echo** | | **2D STE** | |  |
| --- | --- | --- | --- | --- | --- | --- | --- | --- | --- | --- | --- | --- | --- | --- | --- | --- | --- | --- | --- | --- |
| Beaumont et al. 2019 | n = 22 soccer | IC | Mixed | 22 / 0 | 12.0±0.3 | | 4.5±1.5 years  11±1 months/year 4±1 sessions/week 9.4±2.4 h/week 1 match/week | n = 22 | 22 / 0 | 11.7±0.3 | | 1.53±1.77 h/week Recreational  physical activity | | Maturity offset | | ASE/ EAVCI Recommendations,  Lang 2015 | | 40-90 frames/s measurement at  endocardial border  EchoPac, GE Healthcare | |  |
| Binnetoglu et al. 2013 | n = 25 basketball  n = 31 soccer  n = 45 swimming n = 16 tennis  n = 23 wresting | IIC IC IIC IC IIIB | Mixed Mixed Endurance Mixed Power | 25 / 0 31 / 0 19 / 26 9 / 7 23 / 0 | 16.2±1.1 15.1±1.1 12.9±1.6 12.2±0.8 15.5±1.5 | | Minimum: 2 years Minimum: 3 h/week | n = 25 | 18 / 7 | 14.7±1.5 | | Sedentary | | Not mentioned | | ASE/ EAVCI Recommendations,  Lang 2015 | | 60-90 frames/s measurement at  endocardial border EchoPac, GE Healthcare ASE/ EAE Recommendations,  Lang 2005 | |  |
| D'Ascenzi et al. 2017* | n = 57 swimming | IIC | Endurance | 57 / 0 | 10.8±0.2 | | 5-6 sessions/week 105-135 min/session | n = 37 | 37 / 0 | 10.2±0.2 | | <2h/day Recreational  physical activity | | Tanner's stages | | ASE/ EACVI/ EAE  Recommendations,  Lang 2015/ Lai 2010 | | 60-90 frames/s measurement at  endocardial border  EchoPac, GE Healthcare Recommendations,  Mor-Avi 2011 | |  |
| D'Ascenzi et al. 2016* | n = 57 swimming | IIC | Endurance | 57 / 0 | 10.8±0.2 | | 5-6 sessions/week 105-135 min/session | n = 37 | 37 / 0 | 10.2±0.2 | | <2h/day Recreational  physical activity | | Tanner's stages | | ASE/ EACVI/ EAE  Recommendations,  Lang 2015/ Lai 2010 | | 60-80 frames/s measurement at  endocardial border  EchoPac, GE Healthcare Recommendations,  Mor-Avi 2011 | |  |
| Rundqvist et al. 2016 | n = 27 endurance | IIC/ IIIC | Endurance | 16 / 11 | 15.5 (13 - 19) | | Minimum: 2 years  Minimum: 5x30 min/week | n = 27 | 16 / 11 | 15.4 (13 -19) | | No regular exercise | | Not mentioned | | ASE/ EAVCI Recommendations,  Lang 2015 | | >40 frames/s measurement at  endocardial border  EchoPac, GE Healthcare | |  |
| Simsek et al. 2013 | n = 24 wrestling n = 22 marathon | IIIB IIC | Power Endurance | 24 / 0 22 / 0 | 16.8±1.9 17.5±2.2 | | Minimum: 2 years  >15 h/week | n = 20 | 20 / 0 | 16.4±1.8 | | Sedentary/  no sports activities | | Not mentioned | | ASE/ EAE Recommendations,  Lang 2005 | | 60-100 frames/s measurement at  endocardial border EchoPac, GE Healthcare | |  |
| Sulovic et al. 2017 | n = 100 dynamic  n = 100 static | IC/ IIC  IIIA | Endurance Power | 100 / 0 100 / 0 | 15.0±1.0 15.4±1.6 | | Minimum: 2 years  5-7 sessions/week Dynamic: 4.1±1.2 years  Static: 3.9±1.0 years | n = 100 | 100 / 0 | 15.2±1.6 | | No competitive sports | | Not mentioned | | Devereux 1986 | | 2D STE was  not assessed | |  |
| Zdravkovic et al. 2010 | n = 94 soccer | IC | Endurance | 94 / 0 | 12.85±0.84 | | 4.6±1.2 years | n = 47 | 47 / 0 | 12.85±0.86 | | <2h/week physical activity | | Not mentioned | | ASE/ EAE Recommendations,  Lang 2005 | | 2D STE was  not assessed | |  |
| * D'Ascenzi 2017 and D'Ascenzi 2016 report data on the same study collective, but different parameters. | | | | | | | | | | | | | | | | | | | | |
|  |  |  |  |  | |  |  |  |  | |  | |  | |  | |  | |  | |
| Overview of studies, involved in this review. Information is given on the study collective, if 2D echocardiography and/ or 2D speckle tracking analysis was applied and according to which recommendations. | | | | | | | | | | | | | | | | | | | | |
|  |  |  |  |  | |  |  |  |  | |  | |  | |  | |  | |  | |

**Supplementary Table 5. Anthropometric data of study participants.**

| **Author** | **Beaumont  et al. 2019** | | **Binnetoglu  et al. 2013** | | | | | | **D'Ascenzi  et al. 2017** | | **D'Ascenzi  et al. 2016** | | **Rundqvist  et al. 2016** | | **Simsek  et al. 2013** | | | **Sulovic  et al. 2017** | | | | **Zdravkovic  et al. 2010** | | | |
| --- | --- | --- | --- | --- | --- | --- | --- | --- | --- | --- | --- | --- | --- | --- | --- | --- | --- | --- | --- | --- | --- | --- | --- | --- | --- |
|  | Athletes | CG | Athletes | | | | | CG | Athletes | CG | Athletes | CG | Athletes | CG | Athletes | | CG | Athletes | | | CG | Athletes | | CG | |
| Sports | Soccer |  | Basketball | Soccer | Swimmer | Wrestling | Tennis |  | Swimmer |  | Swimmer |  | Endurance |  | Running | Wrestling |  | Dynamic | Static |  | | Soccer |  | |  |
| Sex  [m / f] | 22 / 0 | 22 / 0 | 25 / 0 | 31 / 0 | 19 / 26 | 23 / 0 | 9 / 7 | 18 / 7 | 57 / 0 | 37 / 0 | 57 / 0 | 37 / 0 | 16 / 11 | 16 / 11 | 22 / 0 | 24 / 0 | 20 / 0 | 100 / 0 | 100 / 0 | 100 / 0 | | 94 / 0 | 47 / 0 | |  |
| Age  [years] | 12.0 ±0.3 | 11.7 ±0.3 | 16.2 ±1.1 | 15.1 ±1.1 | 12.9 ±1.6 | 15.5 ±1.5 | 12.2 ±0.8 | 14.7 ±1.5 | 10.8 ±0.2 | 10.2 ±0.2 | 10.8 ±0.2 | 10.2 ±0.2 | 15.5  (13-19) | 15.4  (13-19) | 17.5 ±2.2 | 16.8 ±1.9 | 16.4 ±1.8 | 15.0 ±1.5 | 15.4 ±1.6 | 15.2 ±1.6 | | 12.85 ±0.84 | 12.85 ±0.86 | |  |
| Height  [cm] | 151 ±6 | 149 ±7 | 179.2 ±8.7 | 168.8 ±6.2 | 154.6 ±10.6 | 163.2 ±9.3 | 157.5 ±9.9 | 161.8 ±15.4 | 146.3 ±10.9 | 142.2 ±8.3 | 146.3 ±10.9 | 142.2 ±8.3 | n.m. | n.m. | 170 ±5.1 | 164.9 ±6.5 | 166.8 ±7.1 | 169.5 ±7.8 | 167.1 ±10.0 | 166.8 ±13.0 | | 159 ±11 | 163 ±13 | |  |
| Body Mass  [kg] | 40.2 ±5.8 | 44.0 ±11.7 | 72 ±11.4 | 59.8 ±7.11 | 47 ±10.7 | 59.1 ±13.5 | 47.4 ±9.3 | 56.6 ±12.2 | 41.4 ±9.9 | 41.5 ±12.0 | 41.4 ±9.9 | 41.5 ±12.0 | n.m. | n.m. | 63.8 ±5.3 | 68.8 ±5.6 | 66.4 ±5.7 | 59.7 ±7.6 | 60.9 ±10.5 | 59.2 ±10.2 | | 48.3 ±10.6 | 58.3 ±13 | |  |
| SBP  [mmHg] | 100 ±8 | 105 ±13 | 119.4 ±10.4 | 112.6 ±8.6 | 109.6 ±10.1 | 115.7 ±8.2 | 109 ±8 | 113 ±9.2 | 109 ±10 | 104 ±12 | 109 ±10 | 104 ±12 | 120  (95-155) | 115  (105-130) | 105.9 ±12 | 113.5 ±14 | 115.6 ±10 | 115.8 ±7.4 | 119.1 ±7.6 | 117.1 ±7.0 | | 110 ±8 | 108 ±7 | |  |
| DBP  [mmHg] | 61±9 | 61 ±10 | 63.4 ±7.3 | 63.3 ±9.1 | 60.4 ±5.4 | 61.3 ±7.7 | 64.9 ±12 | 64.1 ±9.5 | 70 ±7 | 66 ±8 | 70 ±7 | 66 ±8 | 65  (55-80) | 65  (50-85) | 67.5 ±7.5 | 71 ±4.5 | 70.5 ±6.0 | 69.9 ±5.9 | 72.4 ±9.0 | 68.4 ±0 | | 66 ±8 | 61 ±6 | |  |
| HR  [bpm] | 65 ±8 | 74 ±10 | 72.7 ±13.5 | 74.5 ±15.4 | 77.6 ±15.1 | 72.7 ±13.3 | 85.3 ±11.6 | 74.6 ±10.5 | 72 ±9 | 77 ±12 | 72 ±9 | 77 ±12 | 63  (42-85) | 71  (49-88) | 51.5 ±8.3 | 55.8 ±10.5 | 65.5 ±15.4 | 55.58 ±5.88 | 54.54 ±6.69 | 73.37 ±6.83 | | n.m. | n.m. | |  |
| BSA  [m^2^] | n.m. | n.m. | 1.8 ±0.2 | 1.6 ±0.1 | 1.41 ±0.2 | 1.6 ±0.2 | 1.43 ±0.2 | 1.5 ±0.2 | 1.29 ±0.20 | 1.27 ±0.20 | 1.29 ±0.20 | 1.27 ±0.20 | 1.66  (1.37-1.98) | 1.72  (1.26-2.23) | n.m. | n.m. | n.m. | 1.7 ±0.1 | 1.7 ±0.2 | 1.7 ±0.2 | | 1.45 ±0.2 | 1.61 ±0.24 | |  |
| BMI  [kg/m^2^] | n.m. | n.m. | 22.5 ±2.6 | 20.9 ±1.42 | 19.4 ±2.53 | 21.9 ±3.28 | 18.9 ±2.3 | 21.8 ±6.1 | n.m. | n.m. | n.m. | n.m. | n.m. | n.m. | 22.7 ±2.1 | 25.41 ±2.2 | 24.5 ±1.9 | 20.4 ±1.8 | 21.6 ±2.0 | 21.2 ±1.4 | | 18.75 ±1.92 | 21.59 ±1.86 | |  |

**Supplementary Table 6. Left ventricular structural parameters assessed with 2D transthoracic echocardiography.**

|  | **Beaumont et al. 2019** | | | **Binnetoglu et al. 2013** | | | | | | | | **Rundqvist et al. 2016** | | | | | **Simsek et al. 2013** | | | | **Sulovic et al. 2017** | | | | | | **Zdravkovic et al. 2010** | | | | | | |
| --- | --- | --- | --- | --- | --- | --- | --- | --- | --- | --- | --- | --- | --- | --- | --- | --- | --- | --- | --- | --- | --- | --- | --- | --- | --- | --- | --- | --- | --- | --- | --- | --- | --- |
| **Left ventricular structure** | **athletes** | **CG** | **p** | **athletes** | | | | | | **CG** | **p** | **athletes** | **CG** | | **p** | | **athletes** | | **CG** | **p** | **athletes** | | | **CG** | | **P** | **athletes** | | **CG** | | | **p** | |
|  | n = 22 [22 / 0] Soccer | n = 22 [22 / 0] |  | n = 25  [25 / 0] Basketball | n = 31  [31 / 0] Soccer | | n = 45  [19 / 26] Swimming | n = 23  [23 / 0] Wrestling | n = 16  [9 / 7] Tennis | n = 25  [25 / 0] |  | n = 27  [16 / 11] Endurance | n = 27  [16 / 11] | |  | | n = 22  [22 / 0] Running | n = 24  [24 / 0] Wrestling | n = 20  [22 / 0] |  | n = 100  [100 / 0] Dynamic | n = 100  [100 / 0] Static | n = 100  [100 / 0] | |  | | n = 94  [94 / 0] Soccer | n = 47  [47 / 0] | | |  | | |
| LVEDD [mm] | 42±4 | 44±4 | 0.142 | **48.6±4.5** | | **48.7±3.8** | **44.2±7.5** | **49.1±3.5^f^** | **43.9±3.5** | **47.1±4.6** | **<.001** |  |  | |  | | **52.1±4.4^a^** | **47.2±6.2** | **46.7±4.7** | **0.01** | **49.8±3.3^a^** | **46.3±5.4^b^** | **45.0±2.8** | | **<0.05** | | **46.58±3.91** | **45.12±2.95** | | | **<0.05** | | |
| LVEDD index [mm/m^2^] |  |  |  | **25.8±2.7^a^** | | **29.2±2.8** | **31.61±6.7** | **30.5±4.02** | **30.9±3.8** | **29.9±2.6** | **<.001** | **29  (26-37)** | **26  (22-37)** | | **<0.001** | |  |  |  |  |  |  |  | |  | |  |  | | |  | | |
| LVEDD index [mm/(m^2^)^0.5^] | 37±3 | 38±3 | 0.343 |  | |  |  |  |  |  |  |  |  | |  | |  |  |  |  |  |  |  | |  | | **38.72±2.53** | **35.68±2.54** | | | **<0.001** | | |
| LVESD [mm] | 28±3 | 29±3 | 0.403 |  | |  |  |  |  |  |  |  |  | |  | | **32.4±3.7^a^** | **29.2±5.7** | **29.3±4.3** | **0.047** | **31.6±3.0^a^** | **26.6±3.4^c^** | **28.0±2.9** | | **<0.05** | | **31.09±3.61** | **26.28±3.05** | | | **<0.001** | | |
| LVESD index [mm/m^2^] |  |  |  | **16.7±2^b^** | | **18.87±2.1** | **19.6±3.5** | **19.3±2.9** | **19.6±2.4** | **18.7±2.9** | **0.002** |  |  | |  | |  |  |  |  |  |  |  | |  | |  |  | | |  | | |
| LVESD index [mm/(m^2^)^0.5^] | 25±3 | 25±2 | 0.725 |  | |  |  |  |  |  |  |  |  | |  | |  |  |  |  |  |  |  | |  | | **25.83±2.58** | **20.75±2.30** | | | **<0.001** | | |
| IVS [mm] |  |  |  | **9.4±1.4** | | **9.6±1.5^c^** | **9.2±1.4** | **9±1.3** | **7.6±1.2** | **7.5±0.9** | **<.001** |  |  | |  | | **9.5±1.1** | **11.3±1.2^b^** | **9.4±1.3** | **<0.001** | **9.0±1.2** | **9.1±1.7^c^** | **7.3±1.1** | | **<0.05** | | **8.51±0.99** | **8.27±1.06** | | | **<0.001** | | |
| IVS index [mm/m^2^] |  |  |  | **5±7.7** | | **5.8±0.97** | **6.6±1.2^d^** | **5.6±0.7** | **5.3±0.9** | **4.8±0.8** | **<.001** | **3.7  (2.9-4.8)** | **3.1  (2.5-4.9)** | | **<0.001** | |  |  |  |  |  |  |  | |  | |  |  | | |  | | |
| IVS index [mm/(m^2^)^0.5^] |  |  |  |  | |  |  |  |  |  |  |  |  | |  | |  |  |  |  |  |  |  | |  | | **7.08±0.70** | **6.53±0.81** | | | **<0.001** | | |
| LVWT [mm] |  |  |  |  | |  |  |  |  |  |  |  |  | |  | |  |  |  |  |  |  |  | |  | | 16.71±1.50 | 16.47±2.05 | | | n.s. | | |
| LVWT index [mm/(m^2^)^0.5^] |  |  |  |  | |  |  |  |  |  |  |  |  | |  | |  |  |  |  |  |  |  | |  | | **13.09±1.19** | **13.00±1.51** | | | **<0.001** | | |
| LVPWT [mm] |  |  |  |  | |  |  |  |  |  |  |  |  | |  | | **10.2±0.9^a^** | **11.1±1.1^b^** | **9.4±0.9** | **<0.001** | **8.6±1.0** | **8.9±1.6^c^** | **7.0±1.1** | | **<0.05** | | 8.19±0.77 | 8.19±1.11 | | | n.s. | | |
| LVPWT index [mm/m^2^] |  |  |  | **5.2±0.6** | | **5.7±1.12** | **6.6±1.2^e^** | **5.5±0.8** | **5.3±1.2** | **4.9±0.7** | **<.001** | **4.5  (3.8-6.7)** | **4.0  (3.2-4.9)** | | **<0.001** | |  |  |  |  |  |  |  | |  | |  |  | | |  | | |
| LVPWT index [mm/(m^2^)^0.5^] |  |  |  |  | |  |  |  |  |  |  |  |  | |  | |  |  |  |  |  |  |  | |  | | **6.82±0.73** | **6.46±0.80** | | | **<0.001** | | |
| MWT [mm] | 6.0±0.4 | 6.1±0.5 | 0.754 |  | |  |  |  |  |  |  |  |  | |  | |  |  |  |  |  |  |  | |  | |  |  | | |  | | |
| MWT index [mm/(m^2^)^0.5^] | 5.3±0.4 | 5.3±0.5 | 0.769 |  | |  |  |  |  |  |  |  |  | |  | |  |  |  |  |  |  |  | |  | |  |  | | |  | | |
| RWT | 0.29±0.04 | 0.28±0.04 | 0.387 |  | |  |  |  |  |  |  |  |  | |  | |  |  |  |  | **0.35±0.01^a^** | **0.43±0.01^c^** | **0.30±0.01** | | **<0.05** | | 0.36±0.04 | 0.36±0.04 | | | n.s. | | |
| LVM [g] | 75±14 | 82±18 | 0.204 |  | |  |  |  |  |  |  |  |  | |  | | **202±24** | **208±30** | **155±28^c^** | **0.001** | **154.6±35.2** | **148.1±57.0^c^** | **99.7±28.4** | | **<0.05** | | 160.14±33.14 | 149.49±34.01 | | | n.s. | | |
| LVM index [g/m^2^] |  |  |  | **86.9±15.1** | | **94.9±15.1^c^** | **91.6±16.6** | **94.4±14** | **74±16.7** | **76.8±15.7** | **<.001** | **67  (48-100)** | **45  (37-73)** | | **<0.001** | | **115±15^a^** | **119±18** | **88±14^c^** | **0.001** |  |  |  | |  | | **109.79±15.04** | **92.84±18.29** | | | **<0.001** | | |
| LVM index [g/(m^2^)^1.5^] |  |  |  |  | |  |  |  |  |  |  |  |  | |  | |  |  |  |  |  |  |  | |  | | **91.55±13.86** | **73.73±16.54** | | | **<0.001** | | |
| LVM index [g/m^2.7^] | 25±5 | 28±7 | 0.051 | **34.1±7.2** | | **38.6±6.8** | **39.8±7.1** | **40.9±7.4^g^** | **31.4±8.2** | **34.2±11.9** | **<.001** |  |  | |  | |  |  |  |  | **36.9±7.3^a^** | **41.6±11.7^c^** | **24.8±5.0** | | **<0.05** | |  |  | | |  | | |
| LV length [mm] | 76±6 | 74±5 | 0.316 |  | |  |  |  |  |  |  |  |  | |  | |  |  |  |  |  |  |  | |  | |  |  | | |  | | |
| LV length index [mm/(m^2^)^0.5^] | 67±5 | 64±5 | 0.14 |  | |  |  |  |  |  |  |  |  | |  | |  |  |  |  |  |  |  | |  | |  |  | | |  | | |
| LVEDV [ml] | 75±10 | 69±15 | 0.106 |  | |  |  |  |  |  |  |  |  | |  | | **138±39^a^** | **121±32** | **116±34** | **0.016** |  |  |  | |  | |  |  | | |  | | |
| LVEDV index [ml/m^2^] |  |  |  |  | |  |  |  |  |  |  | **60  (50-80)** | **50  (38-72)** | | **<0.001** | |  |  |  |  |  |  |  | |  | |  |  | | |  | | |
| LVEDV index [ml/(m^2^)^1.5^] | **51±8** | **45±6** | **0.007** |  | |  |  |  |  |  |  |  |  | |  | |  |  |  |  |  |  |  | |  | |  |  | | |  | | |
| LVESV [ml] | 26±4 | 26±8 | 0.696 |  | |  |  |  |  |  |  |  |  | |  | | **45±21^a^** | **40±22** | **41±20** | **0.045** |  |  |  | |  | |  |  | | |  | | |
| LVESV index [ml/m^2^] |  |  |  |  | |  |  |  |  |  |  | **24  (18-31)** | **20  (15-29)** | | **0.002** | |  |  |  |  |  |  |  | |  | |  |  | | |  | | |
| LVESV index [ml/(m^2^)^1.5^] | 18±2 | 17±3 | 0.625 |  | |  |  |  |  |  |  |  |  | |  | |  |  |  |  |  |  |  | |  | |  |  | | |  | | |
|  |  |  |  |  | |  |  |  |  |  |  |  |  | |  | |  |  |  |  |  |  |  | |  | |  |  | | |  | | |
|  |  |  |  | ^a^ p < 0.001 vs. swimming; p = 0.004 vs. wrestling; p = 0.006 vs. tennis; p = 0.02 vs. control ^b^ p = 0.001 vs. swimming; p = 0.024 vs. wrestling; p = 0.022 vs. tennis ^c^ p < 0.001 vs. tennis and control ^d^ p < 0.001 vs. basketball, tennis, and control; p = 0.003 vs. soccer; p = 0.001 vs. wrestling ^e^ p < 0.001 vs. basketball, tennis, and control; p = 0.001 vs. wrestling; p = 0.006 vs. soccer ^f^ p = 0.007 vs. swimming; p = 0.026 vs. tennis ^g^ p = 0.006 vs. tennis | | | | | | | |  | |  | |  | ^a^ p < 0.05 vs. wrestling; p < 0.05 vs. controls ^b^ p < 0.05 vs. running; p < 0.05 vs. controls ^c^ p < 0.05 vs. running; p < 0.05 vs. wrestling | | | | ^a^ p = 0.001 vs. static and controls ^b^ p = 0.044 vs. controls ^c^ p = 0.001 vs. controls | | | | | |  | | |  | | |  |
| Parameter of left ventricular structure assessed in 6 studies. Parameters are displayed in mean values ± standard deviation and median with range in parentheses (Rundqvist et al.), respectively. LVDD = left ventricular end-diastolic diameter. LVDD index = LVDD/ BSA. LVDS = left ventricular end-systolic diameter. LVDS index = LVDS/ BSA. IVSD = interventricular septal thickness in diastole. IVSD index = IVSD/ BSA. LVWT = left ventricular wall thickness. LVWT index = LVWT/ BSA. LVPWT = left ventricular posterior wall thickness. LVPWT index = LVPWT/ BSA. MWT = mean wall thickness. MWT index = MWT/ BSA. RWT = relative wall thickness. LVM = left ventricular mass. LVM index = LVM/ BSA. LV length = left ventricular length. LV length index = LV length/ BSA. LVEDV = left ventricular enddiastolic volume. LVEDV index = LVEDV/ BSA. LVESV = left ventricular endsystolic volume. LVESV index = LVESV/ BSA. | | | | | | | | | | | | | | | | | | | | | | | | | | | | | | | | | |

**Supplementary Table 7. Left ventricular functional parameters assessed with 2D transthoracic echocardiography.**

|  | **Beaumont et al. 2019** | | | | | | **Binnetoglu et al. 2013** | | | | | | | | | | | | | | | | **Rundqvist et al. 2016** | | | | | | | | **Simsek et al. 2013** | | | | | | | | | | | | | **Sulovic et al. 2017** | | | | | | | | | | | | | **Zdravkovic et al. 2010** | | | | | | | | | | | | |  |
| --- | --- | --- | --- | --- | --- | --- | --- | --- | --- | --- | --- | --- | --- | --- | --- | --- | --- | --- | --- | --- | --- | --- | --- | --- | --- | --- | --- | --- | --- | --- | --- | --- | --- | --- | --- | --- | --- | --- | --- | --- | --- | --- | --- | --- | --- | --- | --- | --- | --- | --- | --- | --- | --- | --- | --- | --- | --- | --- | --- | --- | --- | --- | --- | --- | --- | --- | --- | --- | --- | --- |
| **Left ventricular function** | **athletes** | **controls** | | | **p** | | **athletes** | | | | | | | | | | | | **CG** | | **p** | | **athletes** | | | | **CG** | | | **p** | **athletes** | | | | | | | | **CG** | | | | **p** | **athletes CG** | | | | | | | | **p** | **p** | | **athletes** | | | | | | **CG** | | | | **p** | |  |  |  |  |
|  | n = 22  [22 / 0] Soccer | n = 22  [22 / 0] | | |  |  | n = 25  [25 / 0] Basketball | | n = 31  [31 / 0] Soccer | | | n = 45  [19 / 26] Swimming | | | n = 23  [23 / 0] Wrestling | | n = 16  [9 / 7] Tennis | | n = 25  [25 / 0] | |  | | n = 27  [16 / 11] Endurance | | | n = 27  [16 / 11] | |  | | | n = 22  [22 / 0] Running | | | n = 24  [24 / 0] Wrestling | | n = 20  [22 / 0] | | | |  | | | | n = 100  [100 / 0] Dynamic | | n = 100  [100 / 0] Static | | | n = 100  [100 / 0] | | |  | n = 94  [94 / 0] Soccer | | | | | | | n = 47  [47 / 0] | | | |  | | |  |  |  |  |
| Tei Index |  |  | | |  | | **0.37±0.08** | | **0.44±0.07** | | | **0.41±0.06** | | | **0.43±0.07** | | **0.50±0.06^a^** | | **0.45±0.10** | | **<.001** | |  | | |  | |  | | |  | | |  | |  | | | |  | | | |  | |  | | |  | | |  |  | | | | | | |  | | | |  | | |  |  |  |  |
| Concentricity  [g/ml]^0.667^ | **4.3±0.7** | **4.9±1.0** | | | **0.017** | |  | |  | | |  | | |  | |  | |  | |  | |  | | |  | |  | | |  | | |  | |  | | | |  | | | |  | |  | | |  | | |  |  | | | | | | |  | | | |  | | |  |  |  |  |
| Sphericity index | **1.8±0.2** | **1.7±0.1** | | | **0.034** | |  | |  | | |  | | |  | |  | |  | |  | |  | | |  | |  | | |  | | |  | |  | | | |  | | | |  | |  | | |  | | |  |  | | | | | | |  | | | |  | | |  |  |  |  |
| SV  [ml] | **49±8** | **43±10** | | | **0.031** | |  | |  | | |  | | |  | |  | |  | |  | |  | | |  | |  | | | **115±32^a^** | | | **95±29** | | **91±26** | | | | **0.038** | | | |  | |  | | |  | | |  |  | | | | | | |  | | | |  | | |  |  |  |  |
| SV index  [ml/(m^2^)^1.0^] | **38±6** | **32±5** | | | **0.002** | |  | |  | | |  | | |  | |  | |  | |  | |  | | |  | |  | | |  | | |  | |  | | | |  | | | |  | |  | | |  | | |  |  | | | | | | |  | | | |  | | |  |  |  |  |
| Q [l/min] | 3.19±0.63 | 3.12±0.67 | | | 0.741 | |  | |  | | |  | | |  | |  | |  | |  | |  | | |  | |  | | |  | | |  | |  | | | |  | | | |  | |  | | |  | | |  |  | | | | | | |  | | | |  | | |  |  |  |  |
| Cardiac index  [l/min/ m^2^]^1.0^ | 2.48±0.46 | 2.36±0.42 | | | 0.391 | |  | |  | | |  | | |  | |  | |  | |  | |  | | |  | |  | | |  | | |  | |  | | | |  | | | |  | |  | | |  | | |  |  | | | | | | |  | | | |  | | |  |  |  |  |
| LV EF  [%] |  |  | | |  | | 64.6±5.1 | | 64.1±4.3 | | | 67.4±6.1 | | | 65.2±5.6 | | 66±3.8 | | 65.9±5.1 | | 0.098 | | **61  (57-67)** | | | **59  (54-67)** | | **0.036** | | | 68±5.5 | | | 68.4±7.2 | | 70.1±3.4 | | | | 0.45 | | | | **80.1±7.2^a^** | | **74.2±3.9^d^** | | | **75.6±5.2** | | | **<0.05** | 68.23±4.36 | | | | | | | 68.49±4.72 | | | | n.s. | | |  |  |  |  |
| FS  [%] |  |  | | |  | | 35.1±4.2 | | 35±3.5 | | | 37±5.1 | | | 35.9±4.8 | | 36.2±2.9 | | 36.3±4 | | 0.412 | |  | | |  | |  | | |  | | |  | |  | | | |  | | | |  | |  | | |  | | |  | 38.01±3.84 | | | | | | | 38.36±3.95 | | | | n.s. | | |  |  |  |  |
| S'  [cm/s] |  |  | | |  | |  | |  | | |  | | |  | |  | |  | |  | | 7.5  (5.3-9.3) | | | 7.8  (6.4-11.3) | | 0.13 | | |  | | |  | |  | | | |  | | | |  | |  | | |  | | |  |  | | | | | | |  | | | |  | | |  |  |  |  |
| MAPSE  [cm] |  |  | | |  | |  | |  | | |  | | |  | |  | |  | |  | |  | | |  | |  | | |  | | |  | |  | | | |  | | | |  | |  | | |  | | |  |  | | | | | | |  | | | |  | | |  |  |  |  |
| MAPSE index  [mm/m^2^] |  |  | | |  | |  | |  | | |  | | |  | |  | |  | |  | | 8.3  (6.2-10.7) | | | 8.4 (5.6-10.8) | | 0.574 | | |  | | |  | |  | | | |  | | | |  | |  | | |  | | |  |  | | | | | | |  | | | |  | | |  |  |  |  |
| E  [cm/s] |  |  | | |  | | **89 ± 11** | | **92 ± 16** | | | **100 ± 13** | | | **89 ± 15** | | **97 ± 13** | | **95 ± 16** | | **0.026** | | 102  (76-125) | | | 96  (72-117) | | 0.08 | | | 101±18 | | | 99±16 | | 106±25 | | | | 0.497 | | | | **125±10^b^** | | **117±13^d^** | | | **123±10** | | | **<0.05** |  | | | | | | |  | | | |  | | |  |  |  |  |
| A  [cm/s] |  |  | | |  | | **53 ± 10** | | **52 ± 10** | | | **52 ± 10** | | | **47 ± 10^b^** | | **56 ± 13** | | **60 ± 10** | | **0.043** | | 42  (31-56) | | | 43  (30-68) | | 0.277 | | | 56±16 | | | 57±14 | | 65±17 | | | | 0.115 | | | | **93±0.9^c^** | | **90±0.9^d^** | | | **95±0.7** | | | **<0.05** |  | | | | | | |  | | | |  | | |  |  |  |  |
| E/A |  |  | | |  | | 1.7 ± 0.4 | | 1.8 ± 0.4 | | | 1.9 ± 0.4 | | | 1.9 ± 0.4 | | 1.75 ± 0.28 | | 1.7 ± 0.4 | | 0.06 | | **2.5  (1.8-3.6)** | | | **2.1  (1.3-3.5)** | | **0.046** | | | 1.81±0.15 | | | 1.74±0.18 | | 1.65±0.21 | | | | 0.085 | | | | 1.35±0.11 | | 1.33±0.13 | | | 1.34±0.10 | | | n.s. |  | | | | | | |  | | | |  | | |  |  |  |  |
| DT  [ms] |  |  | | |  | | **100.2 ± 11.1** | | **102.9 ± 24.7** | | | **111.7 ± 14.5** | | | **102.2 ± 14.6** | | **136.56 ± 26.39^c^** | | **101.8 ± 18.6** | | **<0.001** | |  | | |  | |  | | |  | | |  | |  | | | |  | | | |  | |  | | |  | | |  |  | | | | | | |  | | | |  | | |  |  |  |  |
| E'  [cm/s] |  |  | | |  | | 19 ± 3 | | 19 ± 3 | | | 18 ± 2 | | | 20 ± 3 | | 19 ± 3 | | 19 ± 3 | | 0.255 | | 14  (12-18) | | | 13  (10-15) | | 0.452 | | |  | | |  | |  | | | |  | | | |  | |  | | |  | | |  |  | | | | | | |  | | | |  | | |  |  |  |  |
| A'  [cm/s] |  |  | | |  | | 7 ± 1 | | 6 ± 1 | | | 6 ± 1 | | | 6 ± 1 | | 9 ± 13 | | 6 ± 10 | | 0.547 | | 4.7  (2.5-7.2) | | | 4.9  (3.2-8.2) | | 0.489 | | |  | | |  | |  | | | |  | | | |  | |  | | |  | | |  |  | | | | | | |  | | | |  | | |  |  |  |  |
| E/E' |  |  | | |  | |  | |  | | |  | | |  | |  | |  | |  | | 7.6  (5.8-9.6) | | | 7.5  (5.7-9.5) | | 0.477 | | |  | | |  | |  | | | |  | | | |  | |  | | |  | | |  |  | | | | | | |  | | | |  | | |  |  |  |  |
| E'/A' |  |  | | |  | | 2.67 ± 0.56 | | 3.22 ± 0.99 | | | 2.77 ± 0.61 | | | 3.14 ± 0.92 | | 3.08 ± 0.63 | | 2.95 ± 0.93 | | 0.06 | |  | | |  | |  | | |  | | |  | |  | | | |  | | | |  | |  | | |  | | |  |  | | | | | | |  | | | |  | | |  |  |  |  |
| S' septal annulus  [cm/s] |  |  | | |  | |  | |  | | |  | | |  | |  | |  | |  | |  | | |  | |  | | | 9.2±1.4 | | | 9.8±1.2 | | 9.4±1.5 | | | | 0.377 | | | |  | |  | | |  | | |  |  | | | | | | |  | | | |  | | |  |  |  |  |
| E' septal annulus  [cm/s] |  |  | | |  | |  | |  | | |  | | |  | |  | |  | |  | |  | | |  | |  | | | 15.3±2.8 | | | 14.7±2.5 | | 14.3±2.7 | | | | 0.43 | | | |  | |  | | |  | | |  |  | | | | | | |  | | | |  | | |  |  |  |  |
| A' septal annulus  [cm/s] |  |  | | |  | |  | |  | | |  | | |  | |  | |  | |  | |  | | |  | |  | | | 8.2±2.1 | | | 9.3±2.7 | | 9.4±2.1 | | | | 0.177 | | | |  | |  | | |  | | |  |  | | | | | | |  | | | |  | | |  |  |  |  |
| S' lateral annulus  [cm/s] |  |  | | |  | |  | |  | | |  | | |  | |  | |  | |  | |  | | |  | |  | | | **8.8±2.3^a^** | | | **10.4±1.8** | | **9.9±2.1** | | | | **0.045** | | | |  | |  | | |  | | |  |  | | | | | | |  | | | |  | | |  |  |  |  |
| E' lateral annulus  [cm/s] |  |  | | |  | |  | |  | | |  | | |  | |  | |  | |  | |  | | |  | |  | | | 17.7±5.1 | | | 17.1±3.8 | | 18.2±3.1 | | | | 0.685 | | | |  | |  | | |  | | |  |  | | | | | | |  | | | |  | | |  |  |  |  |
| A' lateral annulus  [cm/s] |  |  | | |  | |  | |  | | |  | | |  | |  | |  | |  | |  | | |  | |  | | | 7.8±2.3 | | | 8.5±3.4 | | 8.6±2.4 | | | | 0.56 | | | |  | |  | | |  | | |  |  | | | | | | |  | | | |  | | |  |  |  |  |
|  |  |  | | |  | |  | |  | | |  | | |  | |  | |  | |  | |  | | |  | |  | | |  | | |  | |  | | | |  | | | |  | |  | | |  | | |  |  | | | | | | |  | | | |  | | |  |  |  |  |
|  |  |  | | |  | | ^a^ p < 0.001 vs. basketball and swimming  ^b^ p = 0.037 vs. control  ^c^ p < 0.001 vs. basketball, soccer, swimming, wrestling, and controls | | | | | | | | | | | | | | | | |  | |  | |  | | | ^a^ p < 0.05 vs. wrestling and controls | | | | | | | | | | | | | ^a^ p = 0.001 vs. static; p = 0.036 vs. controls  ^b^ p = 0.001 vs. static  ^c^ p = 0.004 vs. static  ^d^ p = 0.001 vs. controls | | | | | | | | | | | | |  | | | | | |  | | | |  |  |  |  |
|  |  |  | | |  | |  | | |  | | |  | |  | |  | |  | |  | | |  | |  | |  | | |  | | | |  | | |  | | | |  | |  | | | |  | | |  | | | | |  | | |  | | | |  | | | |  |  |  |  |
|  |  |  | | |  | |  | | |  | | |  | |  | |  | |  | |  | | |  | |  | |  | | |  | | | |  | | |  | | | |  | |  | | | |  | | |  | | | | |  | | |  | | | |  | | | |  |  |  |  |
| Parameters of left ventricular function assessed in 6 studies. Parameters are displayed in mean values ± standard deviation and median with range in parentheses (Rundqvist et al.), respectively. Tei Index = left ventricular index of myocardial performance. SV = stroke volume. SV index = SV/ BSA. Q = cardiac output. Cardiac index = Q/ BSA. LV EF = left ventricular ejection fraction. FS = fractional shortening. S' = systolic peak velocity at mitral valve. MAPSE = mitral annulus plane systolic excursion. MAPSE index = MAPSE/ BSA. E = early diastolic peak velocity. A = late diastolic peak velocity. E/A = E/A ratio. DT = deceleration time. E' = early diastolic peak velocity at mitral valve. A' = late diastolic peak velocity at mitral valve. E/E' = E/E' ratio. E'/A' = E'/A' ratio. | | | | | | | | | | | | | | | | | | | | | | | | | | | | | | | | | | | | | | | | | | | | | | | | | | | | | | | | | | | | | | | | | | | | | | |
|  | | |  |  | |  | |  | | |  | | |  | |  | |  | |  | |  | | |  | | | |  | | |  |  | | | |  | | | |  | | | |  | |  | | |  | | | |  | | | |  | | | |  | | | |  | | |  | |
|  | | |  |  | |  | |  | | |  | | |  | |  | |  | |  | |  | | |  | | | |  | | |  |  | | | |  | | | |  | | | |  | |  | | |  | | | |  | | | |  | | | |  | | | |  | | |  | |

**Supplementary Table 8. Right ventricular structure parameters assessed with 2D transthoracic echocardiography.**

|  | **D'Asecenzi et al. 2017** | | | **Rundqvist et al. 2016** | | |
| --- | --- | --- | --- | --- | --- | --- |
| **Right ventricular structure** | **athletes** | **controls** | **p** | **athletes** | **controls** | **p** |
|  | n = 57 [57 / 0] Swimmer | n = 37 [37 / 0] |  | n = 27 [16 / 11] Endurance | n = 27 [16 / 11] |  |
| RVOT PLAX [mm] | **23.4±2.3** | **22.3±2.4** | **0.035** |  |  |  |
| RVOT PLAX index [mm/m^2^] | 18.5±2.7 | 16.8±5.0 | 0.18 |  |  |  |
| RVOT PSAX [mm] | 25.5±4.2 | 24.5±3.2 | 0.23 |  |  |  |
| RVOT PSAX index [mm/m^2^] | 20.1±3.6 | 19.0±4.2 | 0.95 | **16 (14-23)** | **15 (11-19)** | **0.006** |
| RVOT distal diameter [mm] | **22.2±3.1** | **20.2±2.8** | **0.005** |  |  |  |
| RVOT distal diameter index [mm/m^2^] | 17.3±3.1 | 16.4±2.0 | 0.15 |  |  |  |
| RV basal diameter [mm] | **31.4±3.2** | **29.5±2.7** | **0.007** |  |  |  |
| RV basal diameter index [mm/m^2^] | 24.9±4.1 | 23.6±3.0 | 0.15 | **23 (19-28)** | **20 (17-28)** | **<.001** |
| RV mid-cavity diameter [mm] | **29.1±3.3** | **24.5±2.8** | **<0.0001** |  |  |  |
| RV mid-cavity diameter index [mm/m^2^] | 23.1±4.2 | 19.4±2.7 | 0.07 |  |  |  |
| RV end-diastolic area [cm^2^] | **14.7±2.8** | **13.8±2.8** | **0.05** |  |  |  |
| RV end-diastolic area index [cm^2^/m^2^] | 11.5±1.8 | 10.6±2.3 | 0.12 | **15 (11-21)** | **13 (9-17)** | **<0.001** |
| RV end-systolic area [cm^2^] | **8.3±1.7** | **7.2±1.9** | **0.002** |  |  |  |
| RV end-systolic area index [cm^2^/m^2^] | **6.6±1.5** | **5.5±1.6** | **0.003** |  |  |  |
|  |  |  |  |  |  |  |
| Parameters of right ventricular structure assessed in 2 studies. Parameters are displayed as mean values ± standard deviation (D'Ascenzi et al.) or as median and range in parentheses (Rundqvist et al.). RVOT = right ventricular outflow tract. PLAX = parasternal long axis. PSAX = parasternal short axis. RV = right ventricle. All indexed values are indexed to BSA. | | | | | | |

**Supplementary Table 9. Right ventricular function parameters assessed with 2D transthoracic echocardiography.**

|  | **D'Asecenzi et al. 2017** | | | **Rundqvist et al. 2016** | | | |
| --- | --- | --- | --- | --- | --- | --- | --- |
| **Right ventricular function** | **athletes** | **controls** | **p** | **athletes** | **controls** | **p** |  |
|  | n = 57 [57 / 0] Swimmer | n = 37 [37 / 0] |  | n = 27 [16 / 11] Endurance | n = 27 [16 / 11] |  |  |
| TAPSE [cm] | 2.2±0.31 | 2.18±0.26 | 0.63 |  |  |  |  |
| TAPSE index [mm/m^2^] |  |  |  | **12 (7–16)** | **10 (8–16)** | **0.008** |  |
| E/A | 1.6±0.4 | 1.6±0.4 | 0.37 |  |  |  |  |
| E' [cm/s] | 15.9±3.1 | 18.8±3.2 | 0.32 | 11 (7–16) | 11 (8–15) | 0.697 |  |
| A' [cm/s] | 9.5±2.3 | 10.4±2.8 | 0.18 | 6.2 (3.4–10.6) | 5.3 (2.3–10.2) | 0.169 |  |
| E/E' | 4.6±1.4 | 4.2±0.9 | 0.33 |  |  |  |  |
| E'/A' | 1.8±0.6 | 1.7±0.5 | 0.64 |  |  |  |  |
| S' [cm/s] | 14.1±2.1 | 14.2±2.4 | 0.75 | **11 (7–15)** | **10 (8–14)** | **0.031** |  |
| RV FAC [%] | **42.9±7.5** | **48.6±8.2** | **0.007** | 42 (36–49) | 41 (35–48) | 0.119 |  |
|  |  |  |  |  |  |  |  |
| Parameters of right ventricular function assessed in 2 studies. Parameters are displayed as mean values ± standard deviation (D'Ascenzi et al.) or as median and range in parentheses (Rundqvist et al.). TAPSE = tricuspid annulus plane systolic excursion. TAPSE index = TAPSE/ BSA. E/A = early diastolic peak velocity/ late diastolic peak velocity ratio. E' = early diastolic peak velocity at tricuspid valve. A' = late diastolic peak velocity at tricuspid valve. E/E' = E/E' ratio. E'/A' = E'/A' ratio. S' = systolic peak velocity at mitral valve. RV FAC = right ventricular fractional area change. | | | | | | | |

**Supplementary Table 10. Left atrial structure parameters assessed with 2D transthoracic echocardiography.**

|  | **D'Asecenzi et al. 2016** | | | **Rundqvist et al. 2016** | | | **Simsek et al. 2013** | | | | | | | **Sulovic et al. 2017** | | | | | | | **Zdravkovic et al. 2010** | | | | |  |
| --- | --- | --- | --- | --- | --- | --- | --- | --- | --- | --- | --- | --- | --- | --- | --- | --- | --- | --- | --- | --- | --- | --- | --- | --- | --- | --- |
| **Left atrial structure** | **athletes** | **CG** | **p** | **athletes** | **CG** | **p** | **athletes** | | | | **CG** | | **p** | **athletes** | | | | **controls** | | **p** | **athletes** | | **controls** | | **p** |  |
|  | n = 57  [57 / 0] Swimmer | n = 37  [37 / 0] |  | n = 27  [16 / 11] Endurance | n = 27  [16 / 11] |  | n = 22  [22 / 0] Running | n = 24  [24 / 0] Wrestling | | n = 20  [22 / 0] | |  | | n = 100  [100 / 0] Dynamic | n = 100  [100 / 0] Static | | n = 100  [100 / 0] | |  | | n = 94  [94 / 0] Soccer | n = 47  [47 / 0] | |  | |  |
| LA diameter [mm] |  |  |  |  |  |  | **31.8±3.6^a^** | | **29.6±4.8** | | **28.5±4.4** | | **0.046** | 30.5±2.4 | | 29.4±4.1 | | 29.8±3.0 | | n.s. | **27.95±3.43** | | **25.49±3.57** | | **<0.001** |  |
| LA diameter index [mm/m^2^] |  |  |  | **21 (17-27)** | **19 (12-25)** | **0.001** |  | |  | |  | |  |  | |  | |  | |  | **23.18±2.07** | | **20.15±2.87** | | **<0.001** |  |
| LA volume index [ml/m^2^] | 17.2±3.3 | 16.2±2.4 | 0.14 | **27 (21-36)** | **19 (14-31)** | **<0.001** |  | |  | |  | |  |  | |  | |  | |  |  | |  | |  |  |
|  |  |  |  |  |  |  |  | |  | |  | |  |  | |  | |  | |  |  | |  | |  |  |
|  |  |  |  |  |  |  | ^a^ p < 0.05 vs. wrestling and controls | | | | | | |  | |  | |  | |  |  | |  | |  |  |
|  |  |  |  |  |  |  |  | |  | |  | |  |  | |  | |  | |  |  | |  | |  |  |
| Parameters of left atrial structure assessed in 5 studies. Parameters are displayed as mean values ± standard deviation or as median and range in parentheses (Rundqvist et al.). LA = left atrium. Indexed parameters are indexed to BSA. | | | | | | | | | | | | | | | | | | | | | | | | | | |

**Supplementary Table 11. Right atrial structure parameters assessed with 2D transthoracic echocardiography.**

|  | **D'Asecenzi et al. 2016** | | | **Rundqvist et al. 2016** | | | |
| --- | --- | --- | --- | --- | --- | --- | --- |
| **Right atrial structure** | **athletes** | **controls** | **p** | **athletes** | **controls** | **p** |  |
|  | n = 57 [57 / 0] Swimmer | n = 37 [37 / 0] |  | n = 27 [16 / 11] Endurance | n = 27 [16 / 11] |  |  |
| RA area [cm^2^] | **10.0±2.2** | **9.0±1.4** | **0.022** |  |  |  |  |
| RA area index [cm^2^/m^2^] |  |  |  | **9.1 (6.6-11.4)** | **7.2 (5.1-8.7)** | **<0.001** |  |
| RA diameter index [mm/m^2^] |  |  |  | **23 (17-28)** | **20 (15-27)** | **0.008** |  |
| RA volume index [ml/m^2^] | **17.6±3.9** | **15.4±2.9** | **0.007** |  |  |  |  |
|  |  |  |  |  |  |  |  |
| Parameters of right atrial structure assessed in 2 studies. Parameters are displayed as mean values ± standard deviation (D'Ascenzi et al.) or as median and range in parentheses (Rundqvist et al.). RA = right atrium. Indexed parametrs are indexed to BSA. | | | | | | | |

**Supplementary Table 12. Left ventricular function assessed with 2D speckle tracking echocardiography.**

|  | **Beaumont et al. 2019** | | |  | **Binnetoglu et al. 2013** | | | | | | | | | | | |  | **Rundqvist et al. 2016** | | | | | **Simsek et al. 2013** | | | | | | | |  |
| --- | --- | --- | --- | --- | --- | --- | --- | --- | --- | --- | --- | --- | --- | --- | --- | --- | --- | --- | --- | --- | --- | --- | --- | --- | --- | --- | --- | --- | --- | --- | --- |
| **2D STE of the  left ventricle** | **athletes** | **CG** | **p** | | **athletes** | | | | | | | | | | **CG** | **p** | | **athletes** | **CG** | | **p** | | **athletes** | | | | **CG** | | | **p** |  |
|  | n = 22  [22 / 0] Soccer | n = 22  [22 / 0] |  |  | n = 25  [25 / 0] Basketball | n = 31  [31 / 0] Soccer | | n = 45  [19 / 26] Swimming | | n = 23  [23 / 0] Wrestling | | n = 16  [9 / 7] Tennis | | n = 25  [25 / 0] | |  | | n = 27  [16 / 11] Endurance | n = 27  [16 / 11] | |  | | n = 22  [22 / 0] Running | n = 24  [24 / 0] Wrestling | n = 20  [22 / 0] | | |  | | |  |
| Longitudinal strain  at 4 chamber view [%] | 20.3±1.6 | 19.6±2.5 | 0.283 | | **14.3±3.7^a^** | **17.5±3** | | **18.3±2.1** | | **17.9±3.3** | | **16.5±3.6** | | **18.4±2.4** | | **<.001** | |  |  | |  | | **21.4±2.6^a^** | **21.7±2^b^** | **17.2±2.3** | | | **<.001** | | |  |
| Longitudinal strain  at 2 chamber view [%] |  |  |  | | **17.1±2.3^b^** | **20±2.3** | | **20±2.5** | | **19.1±2.5** | | **19.3±2.5** | | **18.8±2.8** | | **<.001** | |  |  | |  | | **23.3±2.8^a^** | **22.7±2.6^b^** | **19.4±2.7** | | | **<.001** | | |  |
| Longitudinal strain  at 3 chamber view [%] |  |  |  | | **13.4±3.3^c^** | **16.1±2.6** | | **16.4±2.5** | | **17.5±3** | | **16.5±3.5** | | **17.9±2.7** | | **<.001** | |  |  | |  | | **22.2±2.9^a^** | **21.1±2.2^b^** | **19.1±2.1** | | | **<.001** | | |  |
| Global  longitudinal strain [%] |  |  |  | | **14.9±2.6^c^** | **17.8±1.9** | | **18.2±1.8** | | **18.6±2.4** | | **17.4±2.4** | | **18.4±2.2** | | **<.001** | | 22  (19-25) | 21  (19-27) | | 0.802 | | **22.3±2.2^a^** | **21.8±1.7^b^** | **18.5±2.4** | | | **<.001** | | |  |
| Circumferential strain  at MV base [%] | **22.2±2.5** | **20.2±2.5** | **0.029** | |  |  | |  | |  | |  | |  | |  | |  |  | |  | |  |  |  | | |  | | |  |
| Radial strain  at MV base [%] | 38.3±13.7 | 33.7±15.4 | 0.304 | |  |  | |  | |  | |  | |  | |  | |  |  | |  | |  |  |  | | |  | | |  |
| Circumferential strain  at papillary muscle [%] | **20.1±1.5** | **18.3±2.5** | **0.007** | | **11.8±3.3** | **14.2±3.2** | | **13.8±3** | | **13.4±3.2** | | **12±3.7** | | **12.9±3.1** | | **0.041** | |  |  | |  | |  |  |  | | |  | | |  |
| Radial strain  at papillary muscle [%] | 61.6±15.8 | 62.2±20.9 | 0.918 | | 31.8±14.9 | 42.4±16.2 | | 37.9±14 | | 40.7±18.17 | | 34.6±21 | | 41.9±14.1 | | 0.110 | |  |  | |  | |  |  |  | | |  | | |  |
| Apical rotation [°] | **11.95±5.31** | **7.58±3.55** | **0.005** | |  |  | |  | |  | |  | |  | |  | |  |  | |  | |  |  |  | | |  | | |  |
| Basal rotation [°] | 5.69±3.14 | 5.64±2.65 | 0.959 | |  |  | |  | |  | |  | |  | |  | |  |  | |  | |  |  |  | | |  | | |  |
| Twist [°] | **16.92±7.55** | **12.34±4.99** | **0.035** | |  |  | |  | |  | |  | |  | |  | |  |  | |  | |  |  |  | | |  | | |  |
|  |  |  |  | |  |  | |  | |  | |  | |  | |  | |  |  | |  | |  |  |  | | |  | | |  |
|  |  |  |  | | ^a^ p < 0.001 vs. soccer, swimming, wrestling and controls  ^b^ p < 0.001vs. soccer and swimming  ^c^ p < 0.001vs. soccer, swimming, wrestling, tennis and controls | | | | | | | | | | | | |  | |  | |  | ^a^ p < 0.05 vs. controls  ^b^ p < 0.05 vs. controls | | | | | | | |  |
|  |  |  |  | |  | |  | |  | |  | |  | |  |  | |  | |  | |  |  |  | |  | | |  | |  |
| Overview of 2D STE parameters of the left ventricle in 4 studies. Parameters are displayed as mean values ± standard deviation or as median and range in parentheses (Rundqvist et al.), respectively. | | | | | | | | | | | | | | | | | | | | | | | | | | | | | | |  |

**Supplementary Table 13. Right ventricular function, left and right atrial function assessed with 2D speckle tracking echocardiography.**

|  | **D'Asecenzi et al. 2017** | | | **D'Asecenzi et al. 2016** | | | **Rundqvist et al. 2016** | | |
| --- | --- | --- | --- | --- | --- | --- | --- | --- | --- |
| **2D STE of the right ventricle,  right and left atrium** | **athletes** | **controls** | **p** | **athletes** | **controls** | **p** | **athletes** | **controls** | **p** |
|  | n = 57 [57 / 0] Swimmer | n = 37 [37 / 0] |  | n = 57 [57 / 0] Swimmer | n = 37 [37 / 0] |  | n = 27 [16 / 11] Endurance | n = 27 [16 / 11] |  |
| Longitudinal strain RV  at 4 chamber view [%] | 29.2±5.8 | 31.6±7.0 | 0.063 |  |  |  | 27 (19-34) | 28 (19-33) | 0.25 |
| L-PALS [%] |  |  |  | 61.1±10.2 | 64.2±6.8 | 0.15 |  |  |  |
| L-PACS [%] |  |  |  | 16.4±4.8 | 15.5±4.1 | 0.41 |  |  |  |
| LA total strain [%] |  |  |  |  |  |  | 39 (31-53) | 38 (28-52) | 0.873 |
| R-PALS [%] |  |  |  | 53.1±11.7 | 58.2±12.6 | 0.071 |  |  |  |
| R-PACS [%] |  |  |  | 16.9±4.6 | 11.8±6.2 | 0.58 |  |  |  |
|  |  |  |  |  |  |  |  |  |  |
|  |  |  |  |  |  |  |  |  |  |
|  |  |  |  |  |  |  |  |  |  |
|  |  |  |  |  |  |  |  |  |  |
| Overview of 4 studies reporting 2D speckle tracking echocardiography parameters of the right ventricle. Parameters are displayed in mean values ± standard deviation (D'Ascenzi et al.) and median with range in parentheses (Rundqvist et al.), respectively. L-PALS = left peak atrial longitudinal strain. L-PACS = left peak atrial contraction strain. R-PALS = right peak atrial longitudinal strain. R-PACS = right peak atrial contraction strain. | | | | | | | | | |
|  |  |  |  |  |  |  |  |  |  |
